# Oleic acid-induced NOX4 is dependent on ANGPTL4 expression to promote human colorectal cancer metastasis

**DOI:** 10.7150/thno.44744

**Published:** 2020-05-30

**Authors:** Chih-Jie Shen, Kwang-Yu Chang, Bo-Wen Lin, Wei-Ting Lin, Che-Min Su, Jhih-Peng Tsai, Yu-Han Liao, Liang-Yi Hung, Wen-Chang Chang, Ben-Kuen Chen

**Affiliations:** 1 Department of Pharmacology, College of Medicine, National Cheng Kung University, Tainan 701, Taiwan, ROC.; 2 Graduate Institute of Medical Science, College of Medicine, Taipei Medical University, Taipei 110, Taiwan, ROC.; 3 National Institute of Cancer Research, National Health Research Institutes, Tainan 701, Taiwan, ROC.; 4 Department of Internal Medicine, National Cheng Kung University Hospital, College of Medicine, National Cheng Kung University, Tainan 701, Taiwan, ROC.; 5 Department of Surgery, National Cheng Kung University Hospital, College of Medicine, National Cheng Kung University, Tainan 701, Taiwan, ROC.; 6 Department of Biotechnology and Bioindustry Sciences, College of Bioscience and Biotechnology, National Cheng Kung University, Tainan 701, Taiwan, ROC.; 7 Graduate Institute for Cancer Biology and Drug Discovery, College of Medical Science and Technology, Taipei Medical University, Taipei 110, Taiwan, ROC.

**Keywords:** colorectal cancer, oleic acid, ANGPTL4, NOX4, metastasis

## Abstract

**Background:** Colorectal cancer (CRC) progression and related mortality are highly associated with metabolic disorders. However, the molecular mechanism involved in the regulation of hyperlipidemia-associated CRC metastasis remains unclear. This study aimed to investigate the effects of angiopoietin-like 4 (ANGPTL4) on NADPH oxidase 4 (NOX4) expression and reactive oxygen species (ROS) production, which might provide new targets for improving outcomes in patients with hyperlipidemia-associated CRC metastasis.

**Methods:** The clinical relevance of relationship between NOX4 expression and ANGPTL4 was examined in CRC patients by the Oncomine and TCGA data set. Expressions of NOX4, epithelial-mesenchymal transition (EMT) markers, and gene regulation of NOX4 in free fatty acids (FFAs)-treated CRC cells were determined. The FFAs-triggered metastatic ability of CRC cells under treatments of antioxidants or knockdown of NOX4, ANGPTL4, and MMPs was evaluated *in vitro* and *in vivo*. In addition, effects of antioxidants and depletion of metastasis-associated molecules on the correlation between ROS production and FFAs-promoted CRC metastasis were also clarified.

**Results:** In this study, we found that the induction of NOX4, followed by the increased ROS was essential for oleic acid (OA)-promoted CRC cell metastasis. The depletion of ANGPTL4 significantly inhibited c-Jun-mediated transactivation of NOX4 expression, accompanied with reduced levels of ROS, MMP-1, and MMP-9, resulting in the disruption of OA-promoted CRC cell metastasis. Moreover, knockdown of ANGPTL4, NOX4, MMP-1, and MMP-9 or the treatment of antioxidants dramatically inhibited circulating OA-enhanced tumor cell extravasation and metastatic seeding of tumor cells in lungs, indicating that the ANGPTL4/NOX4 axis was critical for dyslipidemia-associated tumor metastasis.

**Conclusion:** The coincident expression of NOX4 and ANGPTL4 in CRC tumor specimens provides the insight into the potential therapeutic targets for the treatment of dyslipidemia-associated CRC metastasis.

## Introduction

Colorectal cancer (CRC) is the second leading type of cancer worldwide and is a common malignant tumor of the digestive system 1. The five-year survival rate is more than 90% for patients with early-stage CRC. However, late-stage CRC with distant metastasis has a 10-15% survival rate for cancer patients 2. Metastatic CRC remains a therapeutic challenge, which can partially be explained by disease heterogeneity development over time and at the time of occurrence 3. Thus, clarification of the molecular mechanisms underlying CRC metastasis has been one of the major objectives of cancer research. Extracellular signals in the cancer microenvironment have been implicated in the epithelial-to-mesenchymal transition (EMT) program that promotes cancer progression. For example, carcinoma-associated fibroblast promotes stemness of CRC by transferring exosomal long non-coding RNA H19 4. The TGF-β signaling pathway is an essential driver of EMT in CRC 5. The activation of Ezrin/NF-κB by epidermal growth factor (EGF) triggers CRC cells to undergo EMT 6. Proinflammatory cytokines, including IL-8 and IL-6, have been reported to induce EMT via PI3K/AKT-ERK1/2 crosstalk and STAT3/Fra-1 signaling, respectively, to mediate CRC metastasis 7,8. Intriguingly, a high-fat diet (HFD) was found to initiate EMT through the MAPK/ERK and PI3K/AKT/mTOR signaling cascades in mouse xenograft model of CRC 9. In addition, metabolic reprogramming through activation of the Glut3-YAP signaling pathway has been shown to support CRC metastasis 10. These findings reinforce the connection between metabolic diseases and EMT.

In the clinic, CRC-related mortality can be reduced in patients by using statins to maintain normal cholesterol levels 11. Further evidence suggests that hyperlipidemia caused by an abnormal lifestyle can accelerate the progression of CRC 12. Likewise, CRC patients who consume an HFD accumulate more reactive oxygen species (ROS), which are associated with metastasis progression 13. As a major source of ROS generation, the NADPH oxidase (NOX) family, including NOX1-5 and DUOX1-2, has attracted increasing attention due to its correlation with cancer development and progression 14,15. Among the NOX family members, NOX4 is the most frequently expressed in terms of its relevance in cancer, diabetes, and cardiovascular disease 16,17. NOX4 overexpression has also been found in diverse types of solid tumors, such as prostate cancer, liver cancer, CRC, and melanoma 18-22. Moreover, cycling of hypoxia-induced ROS via NOX4 promotes the expression of MMP-9 in glioblastoma cancer cells 23. These studies indicate that NOX4 plays roles in the regulation of cancer proliferation, tumorigenic transformation, and metastasis. Nevertheless, the molecular mechanisms involved in the regulation of NOX4 and its role in CRC progression and metastasis remain unclear.

Hyperlipidemia is the presence of elevated lipid levels in the blood and is a major risk for cardiovascular disease. The abnormal lipid content can be regulated by N-terminal angiopoietin-like 4 (ANGPTL4), which inhibits clearance of circulating triglycerides via suppression of lipoprotein lipase (LPL) and protects cardiomyocytes against excess fat uptake 24. On the other hand, evidence also suggests that full-length or C-terminal ANGPTL4 functions as a tumor promoter that enhances tumor proliferation and metastasis 25-28. As a consequence, ANGPTL4 is induced under hypoxic conditions with prostaglandin E2 treatment to enhance CRC progression 29. ANGPTL4 also controls ROS levels by activating NOX1 to engage integrin-dependent survival signals, leading cells to mimic anchorage conditions and giving them the ability to bypass anoikis 30. Although ANGPTL4 has been verified to maintain normal cardiovascular functions through its ability to control plasma lipids, its role and molecular mechanisms in regulation of hyperlipidemia-associated CRC metastasis remain incompletely understood.

In this study, we found that fatty acid-regulated ANGPTL4 leads to upregulation of NOX4 expression through the transcription factor c-Jun. Activation of the ANGPTL4/NOX4 axis further elevates ROS levels to promote CRC cell metastasis. Our findings link the interaction between dyslipidemia and cancer metastasis and provide new insight into coincident ANGPTL4 and NOX4 expression as a potential diagnostic biomarker and therapeutic target for CRC therapy.

## Materials and Methods

### Cell culture

Cell lines of colorectal cancer HCT116, SW480, SW620, LoVo, Colo205, DLD-1, and HT-29 were provided from Research Center of Clinical Medicine, National Cheng Kung University Hospital and Bioresource Collection and Research Center (BCRC, Hsinchu City, TW). Cell lines were maintained at 37°C in 10-cm plastic dishes containing 7 ml of culture medium. Each medium was supplemented with 10% fetal bovine serum (Invitrogen, Grand Island, NY, USA), 100 μg/ml streptomycin (Sigma-Aldrich, St Louis, MO, USA) and 100 unit/ ml penicillin (Sigma-Aldrich). SW480 and SW620 were cultured with Leibovitz's L-15 medium (Invitrogen); HCT116 and HT-29 were cultured with McCoy's 5a medium (Invitrogen); LoVo was cultured with F-12K medium; Colo205 and DLD-1 were cultured with RPMI 1640 medium (Invitrogen).

### Reagents and peptide

Full details are available in Supplementary Materials and Methods.

### Transfection of cells with siRNA oligonucleotides or plasmids

Transient transfection of cells with 20 nM siRNA oligonucleotides or plasmids was performed using RNAiMAX or Lipofectamine 2000 (Invitrogen) according to the manufacturer's instruction with slight modifications. The siRNA IDs were as follows, ANGPTL4 (siRNA IDs: HSS181878, HSS181879); NOX4 (siRNA IDs: HSS121312, HSS121313); MMP-1 (siRNA IDs: HSS106609, HSS106610); MMP-3 (siRNA IDs: S8854, S8855); MMP-9 (siRNA IDs: S8862, S8863); c-Jun (siRNA IDs: HSS105641) (Invitrogen); Negative control siRNAs (siRNA IDs: D-001810-10-50) (Dharmacon, Lafayette, CO, USA). For use in transfection, 3.75 μl of RNAiMAX or Lipofectamine 2000 was incubated with siRNA or plasmid in 1.5 ml of Opti-MEM medium (Invitrogen) for 30 min at room temperature. Following the removal of Opti-MEM medium and replacement with 3 ml of fresh culture medium, cells were incubated for an additional 24 h, unless stated otherwise.

### Enzyme-linked immunosorbent assay

Full details are available in Supplementary Materials and Methods.

### Luciferase assay

Full details are available in Supplementary Materials and Methods.

### Zymography assay

Full details are available in Supplementary Materials and Methods.

### Lentivirus knockdown assay

Full details are available in Supplementary Materials and Methods.

### Reverse transcription polymerase chain reaction (RT-PCR) and primer sets

Full details are available in Supplementary Materials and Methods.

### Real-time quantitative PCR

Full details are available in Supplementary Materials and Methods.

### Western blotting

Full details are available in Supplementary Materials and Methods.

### Boyden chamber and transwell assays

Both assays were performed using Millicell™ hanging cell culture inserts (polyethylene terephthalate (PET) membranes with 8 µm pores) (Millipore, Bedford, MA, USA). For the invasion assay, 5 × 10^5^ cells were plated in serum-free medium containing with or without 200 μM OA and placed in the upper chamber on a diluted matrigel-coated membrane (Millipore), while the lower chamber was filled with 2% FBS medium. After incubation for 72 h, the cells in the upper chamber were removed and the invaded cells at the bottom of the PET membrane were fixed with 4% paraformaldehyde and stained with 0.5% crystal violet (Sigma-Aldrich). Crystal violet staining-cells were then solubilized with 10% acetic acid and absorbance (OD, 595 nm) and were measured in a microplate reader.

### Estimation of ROS levels by flow cytometry

Cells (1 × 10^5^) were seeding in the 3.5 cm dish. After treatment of oleic acid, the cells were stained with 100 nM carboxyl-H2DCFDA or mitoSOX for 30 min in PBS. Then the cells were recovered on 10% FBS medium for 15 min. Cells were then trypsinized and resuspended in PBS. The DCFDA or mitoSOX emission was measured at green channel (FL1) or red channel (FL2), respectively, on a BD Accuri™ C6 Plus (BD Biosciences, San Jose, CA, USA) flow cytometer. Ten thousand events were collected for each sample.

### CFSE proliferation assay

Full details are available in Supplementary Materials and Methods.

### Quantitative estimation of H_2_O_2_ concentration

Full details are available in Supplementary Materials and Methods.

### RNA stability assay

Full details are available in Supplementary Materials and Methods.

### Chromatin immunoprecipitation (ChIP)

The protein-DNA complexes were crosslinked using 1% formaldehyde, which was then quenched by adding glycine to a final concentration of 200 mM. Chromatin complexes were sonicated to an average size of 250 bp by a MISONIX Sonicator 3000 (Misonix, Farmingdale, NY, USA). The chromatin was incubated with rabbit monoclonal anti‐c-Jun (#61327) (BD Tranduction Laboratory, Los Angeles, CA) and PureProteomeTM Protein A Magnetic Beads (Millipore) overnight. The immunocomplexes were reverse crosslinked, and the purified DNA was subjected to PCR analysis. The PCR primers were listed as follows: NOX4 specific primers (sense, 5'- TGA ATC AGA TGA TGG TCT ACA CTT G -3'; antisense, 5'- AGT GGT CCA AAG GCT TAA CAT TCC -3'). The PCR products were separated by 2% agarose-gel electrophoresis and visualized with ethidium bromide staining.

### DNA affinity precipitation assay

Full details are available in Supplementary Materials and Methods.

### Plasmid construction

Full details are available in Supplementary Materials and Methods.

### Tumor extravasation assay in an animal model

Tumor metastasis was determined by intravenous (tail vein) injection of cancer cells into 6 weeks old male severe combined immunodeficient (SCID) mice. Briefly, OA was injected into the circulation of mice for 1 h. For the lung extravasation assay, 1,1'-dioctadecyl-3,3,3',3'-tetramethylindocarbocyanine perchlorate (DiI) labelled cells (1 × 10^6^) were resuspended in 100 μl of PBS, then injected into the tail vein of mice. Animals were sacrificed up to 48 h after injection with ethical method. The lungs were fixed with 4% paraformaldehyde, 30% sucrose, and finally embedded in FSC 22 (#3801480) (Leica, CA, USA) for cryosectioned (8 μm). Immunohistochemistry (IHC) was then performed to determine the location of vessel with antibody CD31 (ab28364) (Abcam, MA, USA). Quantification was performed by analysing at least three sections and six fields to determine the number of tumor cells that underwent extravasation. All mice were obtained from the National Cheng Kung University (NCKU) Laboratory Animal Center (Tainan, TWN). The animal study was approved (Approved No. NCKU-IACUC-107-112) by the IACUC of Laboratory Animal Center, Medical College, NCKU.

### Tumor metastasis assay in an animal model

Full details are available in Supplementary Materials and Methods.

### Immunohistochemistry assay

Full details are available in Supplementary Materials and Methods.

### Statistical analysis

Statistical analysis was performed using Prism 6.0 software (GraphPad Software, Inc., San Diego, CA, USA). All data are presented as the mean ± standard errors of the mean (SEM). The student's *t* test was used for the comparison of measureable variants of two groups. Survival curves were calculated using the Kaplan-Meier method, and differences were assessed by log-rank test. A *P*-value less than 0.05 was considered significant and denoted by *. *P* values less than 0.01 and 0.001 are denoted by ** and ***, respectively.

## Results

### OA-induced ROS production occurs through NOX4 induction in CRC cells

**Figure 1:**
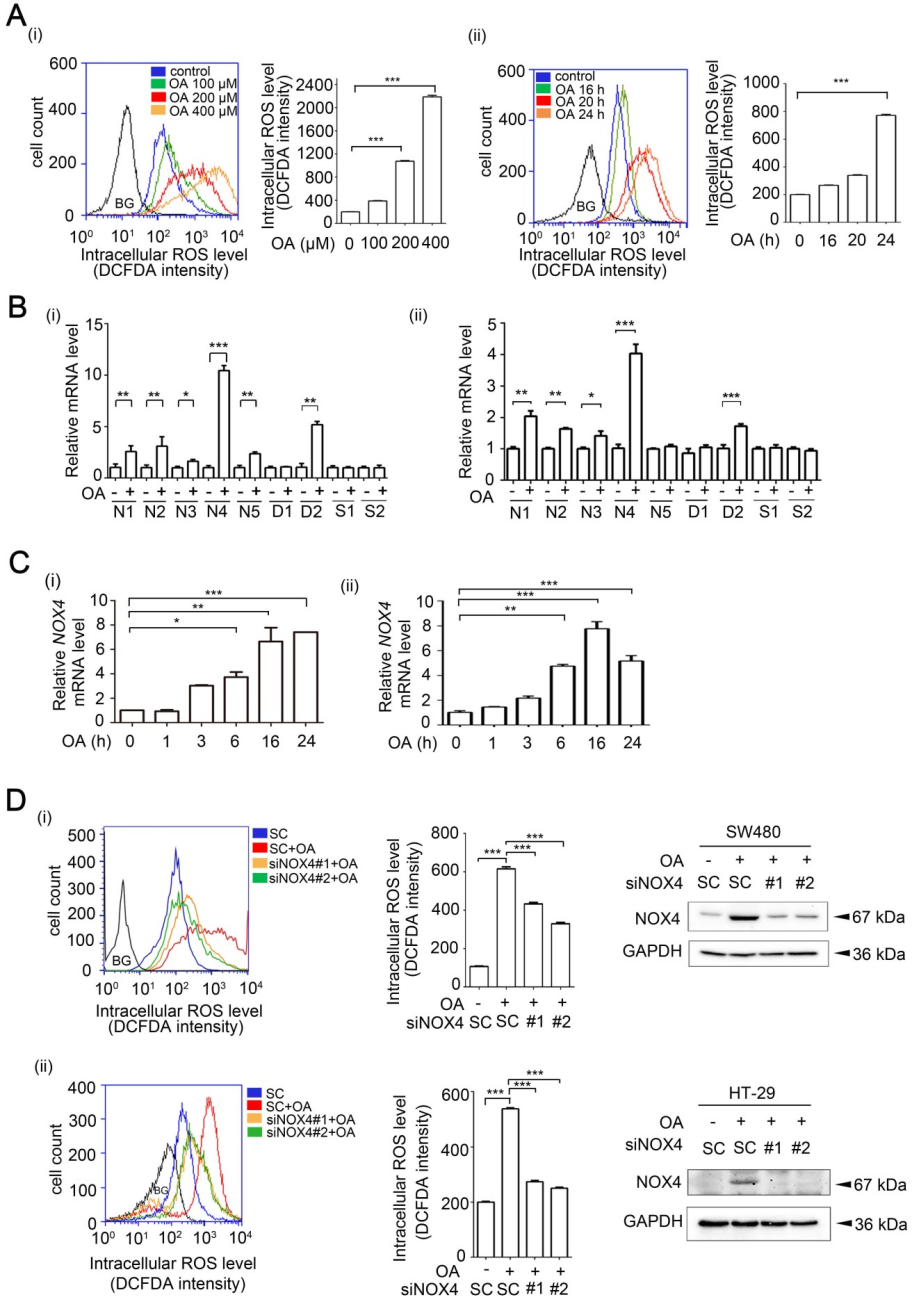
** OA-induced ROS production is dependent on NOX4 expression in CRC cells. (A)** OA-induced ROS levels were examined by flow cytometry analysis with DCFDA staining in SW480 cells treated with OA for various concentrations (i) or periods of time (ii), as indicated. Quantification of ROS levels are shown in columns. BG indicates background. **(B-C)** Real-time quantitative PCR analysis was performed for detecting *NOX1-5* (N1-5), *SOD1-2* (S1-2), *DUOX1-2* (D1-2) mRNA levels in SW480 (i) or HT-29 (ii) cells treated with 200 µM OA for 16 h (B) or the indicated period of time (C). **(D)** ROS levels and NOX4 protein expression were examined by flow cytometry analysis with DCFDA staining and western blotting, respectively. SW480 (i) and HT-29 (ii) cells were transfected with 20 nM scrambled oligonucleotides (SC) or NOX4 siRNA (siNOX4 #1 or #2) for 24 h and then treated with 200 µM OA for 24 h. BG indicates background. The data are presented as the mean ± SEM. *P*-values were determined using a two-tailed Student's *t*-test. *P < 0.05; **P < 0.01; ***P < 0.001 (n=3).

The increase in ROS levels induced by metabolic disorders, such as elevated serum cholesterol, is highly associated with CRC progression 13. To further investigate whether the increase in lipidemia promotes CRC progression, CRC cell lines were treated with free fatty acids (FFAs), such as oleic acid (OA), and then, ROS levels were examined. ROS determination by flow cytometry analysis showed that OA or H_2_O_2_ significantly induced ROS production in CRC cells in a dose- and time-dependent manner (Figure 1A and Figure S1A). In addition, a mitochondria-targeted antioxidant Mito-TEMPO completely inhibited OA-induced mitochondrial ROS production, but partially reduced intracellular ROS levels (Figure S1B), suggesting that OA-induced ROS was partially produced from mitochondria. To identify the enzymes that confer ROS production in OA-treated CRC cells, the expression levels of NOXs, DUOXs and superoxide dismutases (SODs) were examined. The results showed that OA induced the expression of NOXs and DUOX2 but did not induce the expression of SODs in various CRC cell lines (Figure 1B). In addition, the presence of NOX4, the most abundantly expressed OA-induced NOX, was confirmed in several CRC cell lines (Figure S2A), and dynamic changes in NOX4 expression were observed in cells treated with OA for various times (Figure 1C and Figure S2B). Interestingly, increases in ROS levels induced by fatty acids, including OA, linoleic acid (LA), and palmitic acid (PA), were also dramatically reduced in NOX4-depleted cells (Figure 1D and Figure S2C-E). On the other hand, the primary ROS produced in mitochondria is superoxide, which is further converted to hydrogen peroxide by the action of SODs 31. To further confirm the requirement of NOX4 in regulation of OA-promoted ROS production, the levels of hydrogen peroxide were examined in NOX4 knockdown cells. The results showed that NOX4 depletion repressed the OA-induced production of hydrogen peroxide in CRC cells (Figure S3A). Consistent with the OA-induced NOX4 and ROS production, the ROS levels were also increased in cells overexpressing NOX4 and were scavenged by N-acetylcysteine (NAC) (Figure S3B). These results verified that OA-triggered ROS production was at least in part dependent on the expression of NOX4 in CRC cells.

### OA-induced NOX4 promotes the invasion ability of CRC cells through induction of MMP-1 and MMP-9

**Figure 2:**
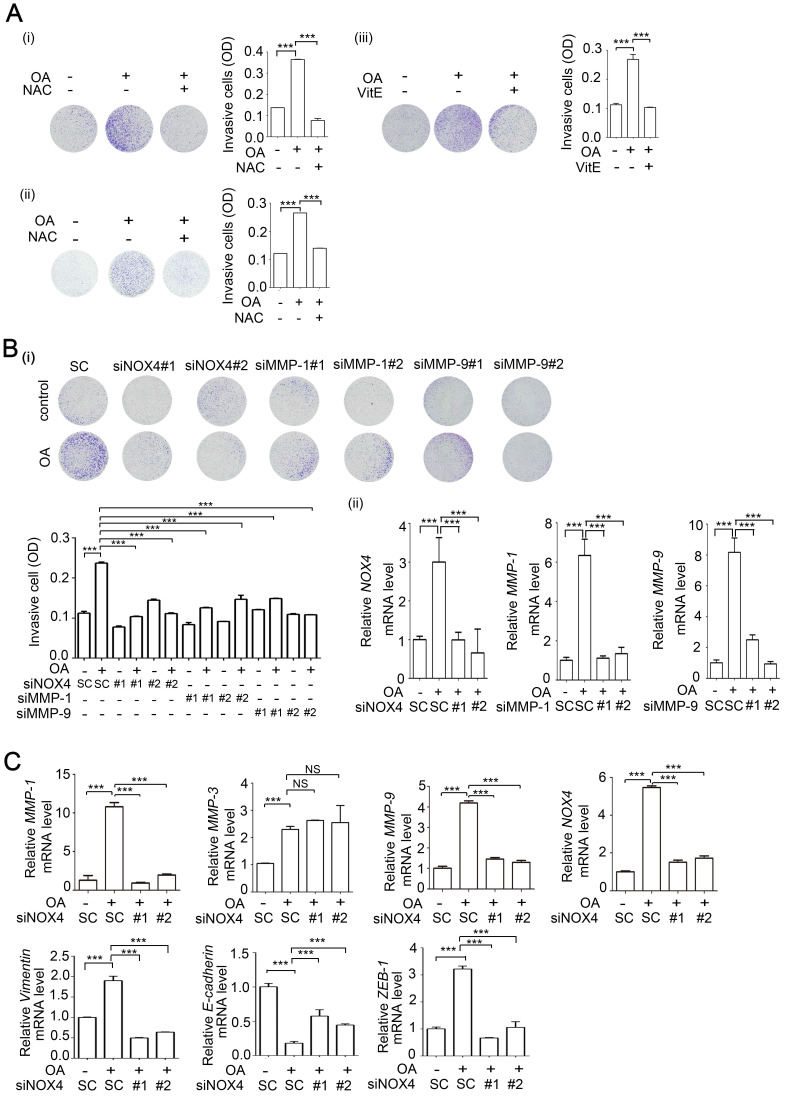
** NOX4 expression is essential for OA-induced invasion ability in CRC cells. (A-B)** Effects of ROS, NOX4, and MMPs on OA-induced cell invasion were analyzed using transwell invasion assays. SW480 (i, iii) and HT-29 cells (ii) were treated with 5 mM NAC or 15 µM Vitamin E (A) or transfected with 20 nM NOX4, MMP-1, and MMP-9 siRNA or scrambled oligonucleotides (SC) **(B)** followed by treatment with 200 µM OA for 72 h. Invading cells were stained with crystal violet and imaged with a microscope, and then solubilized in 10% acetic acid for quantification of invasive levels as shown in columns. The absorbance was measured at a wavelength of 595 nm. The siRNA knockdown efficiency was examined using real-time quantitative PCR (ii). **(C)** Real-time quantitative PCR analysis of *NOX4, MMP-1*, *MMP-3*, *MMP-9, Vimentin, E-cadherin, and ZEB-1* expression in SW480 cells was performed in cells transfected with 20 nM siNOX4 or SC siRNA followed by treatment with 200 µM OA for 16 h. The data are presented as the mean ± SEM. *P*-values were determined using a two-tailed Student's *t*-test. *P < 0.05; **P < 0.01; ***P < 0.001 (n=3).

Our previous study revealed that OA induced HNSCC metastasis 26. We also provided evidence showing that the increase in ROS levels was caused by OA treatment (Figure 1). Here, we studied whether OA can induce CRC cell invasion dependent on NOX4-mediated ROS levels. First, the promotion of cell invasion by fatty acids was verified (Figure S4A-B). The reduction in OA-induced ROS caused by treatment of cells with antioxidants, including NAC and vitamin E, was followed by a decrease in cell invasion (Figure S4C and Figure 2A). In addition, OA-induced MMP-1 and MMP-9 expression was significantly inhibited by antioxidants (Figure S4D). These results suggest that OA-induced CRC cell invasion was dependent on the increase in ROS levels. As shown in Figure 1D, NOX4 was essential for ROS production in OA-treated cells. Therefore, the effect of NOX4 on the OA-induced invasion ability was studied in NOX4 knockdown cells. The results showed that NOX4 depletion significantly attenuated OA-induced cell invasion and led to downregulation of MMP-1, MMP-9, vimentin, and ZEB-1 and upregulation of E-cadherin in NOX4 knockdown cells (Figure 2B-C and Figure S5A-C). Although OA significantly induced MMP-3, no effect of siNOX4 on MMP-3 expression and activation was observed (Figure 2C and Figures S4D, S5B, S5D-E). In addition, the antioxidants had no effect on NOX4 overexpression-induced MMP-3 activity (Figure S5D). To further dissect whether MMPs can mediate OA-enhanced CRC cell invasion, the effects of MMP knockdown on cell invasion were examined. As shown in Figure 2B and Figure 5E, siMMP-1, siMMP-9, and siMMP-3 significantly attenuated the OA-induced invasion ability. These results reveal that MMP-1, -3, and -9 expression is essential for OA-induced cell invasion. However, the OA/NOX4/ROS pathway-induced cell invasion was at least in part dependent on MMP-1 and -9, but not on MMP-3 expression. On the other hand, CRC cell proliferation was not regulated by OA or the depletion of ANGPTL4, NOX4, MMP-1, or MMP-9 genes (Figure S6), suggesting that the OA signaling-regulated cell invasion was independent of cell proliferation. These results reveal that NOX4 expression is essential for OA-promoted cell invasion. To further confirm the role of NOX4 in CRC cell invasion, the effects of elevated NOX4 expression on cell invasion ability and expression of EMT markers were examined. As shown in Figure S7A, overexpression of NOX4 significantly promoted cell invasion and the expression of EMT markers, and the enhancement was repressed by antioxidants. Knockdown of MMP-1 and MMP-9 also reduced NOX4-regulated cell invasion (Figure S7B). In addition, inactivation of NF-κB pathway by expression of a dominant-negative IκB mutant 32 diminished NOX-4-induced MMP-1 and MMP-9 expression (Figure S7C).

### OA-induced ANGPTL4 promotes the invasion ability of CRC cells

**Figure 3:**
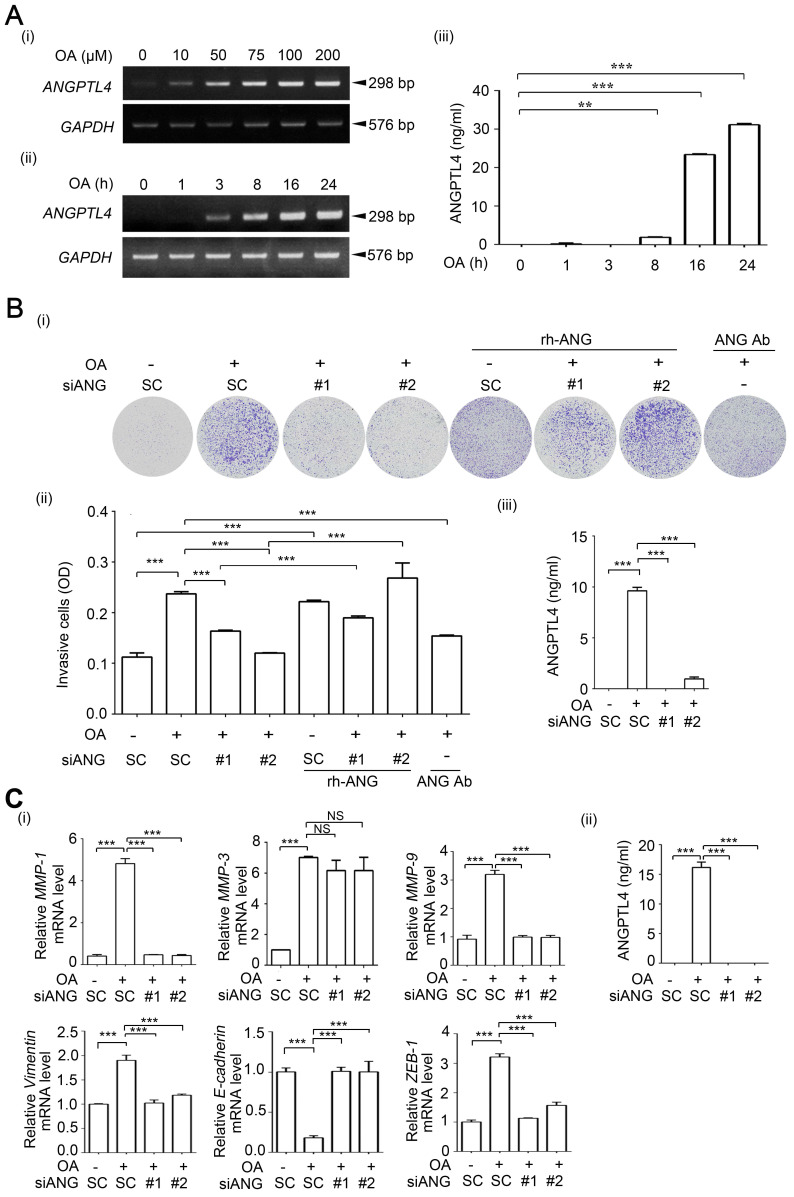
** OA-induced secretion of ANGPTL4 enhances invasion ability in cancer cells. (A)** Semi-quantitative PCR analysis was performed to examine *ANGPTL4* mRNA levels in SW480 cells treated with OA at various concentrations (i) or periods of time (ii), as indicated. ELISAs were performed to asses ANGPTL4 secretion levels (iii). **(B)** Invasion assays were performed using SW480 cells transfected with 20 nM ANGPTL4 siRNA (siANG#1 or #2) or scrambled oligonucleotides (SC) and then treated with 200 µM OA, 1 µg/ml anti-ANGPTL4 antibody (ANG Ab), and 100 ng/ml recombinant human ANGPTL4 (rh-ANG) for 72 h. Invading cells were stained with crystal violet and imaged under a microscope (i), and then solubilized with 10% acetic acid. The absorbance was measured at a wavelength of 595 nm (ii). ELISAs were performed to assess ANGPTL4 secretion in SW480 cells transfected with 20 nM siANGPTL4 or SC siRNA followed by treatment with 200 µM OA for 24 h (iii). **(C)** Real-time quantitative PCR analysis of *MMP-1*, *MMP-3*, *MMP-9, Vimentin, E-cadherin, and ZEB-1* mRNA levels was performed in SW480 cells transfected with 20 nM siANGPTL4 or SC siRNA and then treated with 200 µM OA for 16 h (i). ELISAs were performed to assess ANGPTL4 secretion (ii). The data are presented as the mean ± SEM. *P*-values were determined using a two-tailed Student's *t*-test. *P < 0.05; **P < 0.01; ***P < 0.001. (n=3).

Our previous studies revealed that OA-induced ANGPTL4 contributed to HNSCC metastasis 26. Thus, whether OA-enhanced metastasis in different tumor types is dependent on individual genes, such as ANGPTL4 and NOX4, or if tumor metastasis relies on reciprocal regulation of OA-responsive genes is an interesting pursuit. First, the effects of ANGPTL4 expression on the regulation of OA-induced CRC cell invasion were elucidated. Consistent with our previous studies in HNSCC, OA significantly induced ANGPTL4 expression in a dose- and time-dependent manner in several CRC cell lines (Figure 3A and Figure S8A). An increased activity in ANGPTL4 promoter with PPAR response element (PPRE) suggested that transcriptional activation of the gene was triggered by OA treatment (Figure S8B-C). To further clarify the mechanism involved in transcriptional regulation of the ANGPTL4 gene, OA-activated downstream factors, such as PPARs (Figure S8D), were examined. Although the expression of PTEN and ACOX1a was significantly reduced by inhibition of PPARγ and PPARα, respectively 33, 34, the inhibition of PPARδ, but not PPARα and γ, significantly eliminated OA-induced ANGPTL4 transcriptional activation and gene expression (Figure S8E). In addition, the effect of OA-induced ANGPTL4 on CRC cell invasion was then further investigated. As shown in Figure 3B and S9A, ANGPTL4 knockdown reduced the OA-induced cell invasion ability. The reduction in invasion induced by siANGPTL4 was reversed by treatment with a recombinant ANGPTL4 protein (Figure 3B). The depletion of secreted ANGPTL4 in conditioned medium by anti-ANGPTL4 antibodies also attenuated OA-induced cell invasion ability (Figure 3B). In addition, the OA-regulated expression of EMT markers including MMP-1, MMP-9, vimentin, E-cadherin and ZEB-1, was changed by the depletion of ANGPTL4 in CRC cells (Figure 3C and Figure S9B). These results indicate that induction of ANGPTL4 is also essential for OA-promoted CRC cell invasion.

### OA-induced ANGPTL4 regulates NOX4 expression through activation of c-Jun in CRC cells

**Figure 4:**
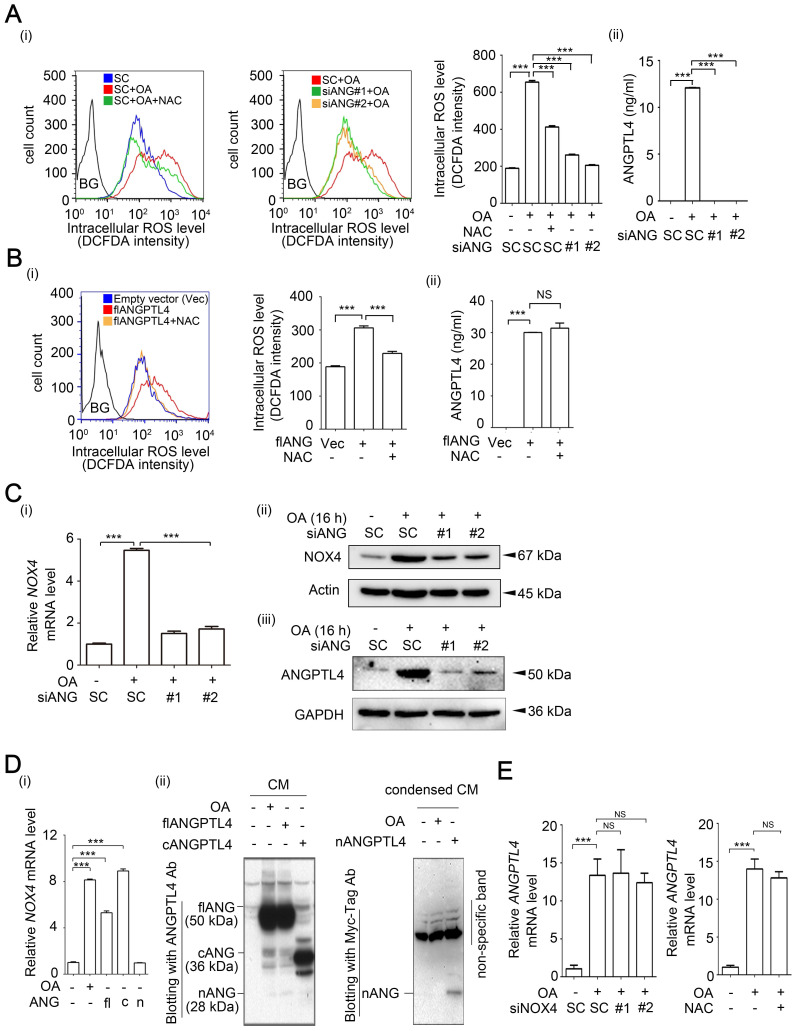
** OA-induced NOX4 and ROS production are regulated by ANGPTL4 in CRC cells. (A-B)** ROS levels were analyzed by flow cytometry analysis with DCFDA staining in SW480 cells transfected with 20 nM siANGPTL4 (siANG#1 and #2), SC siRNA, or expression vector of ANGPTL4 (flANG) and then treated with or without 200 µM OA and 5 mM NAC for 24 h. Quantification of ROS intensities is shown in columns (i). BG indicates background. ELISAs were performed to assess ANGPTL4 secretion in SW480 cells (ii). Vec indicates empty vector. (C-E) Real-time quantitative PCR and immunoblotting analyses for *NOX4* and* ANGPTL4* mRNA and protein levels, respectively were performed in SW480 cells transfected with 20 nM siANGPTL4 (siANG#1 and #2), siNOX4, and SC siRNA **(C, E)**, or expression vectors of full-length (flANG), C-terminal (cANG), and N-terminal (nANG) ANGPTL4 (D), and then treated with or without 200 μM OA and 5 mM NAC for 16 h. Real-time quantitative PCR and western blotting were performed to examine *NOX4* mRNA and ANGPTL4 protein levels, respectively (D). NS indicates not significant. The data are presented as the mean ± SEM. *P*-values were determined using a two-tailed Student's *t*-test. ***P < 0.001 (n=3).

**Figure 5:**
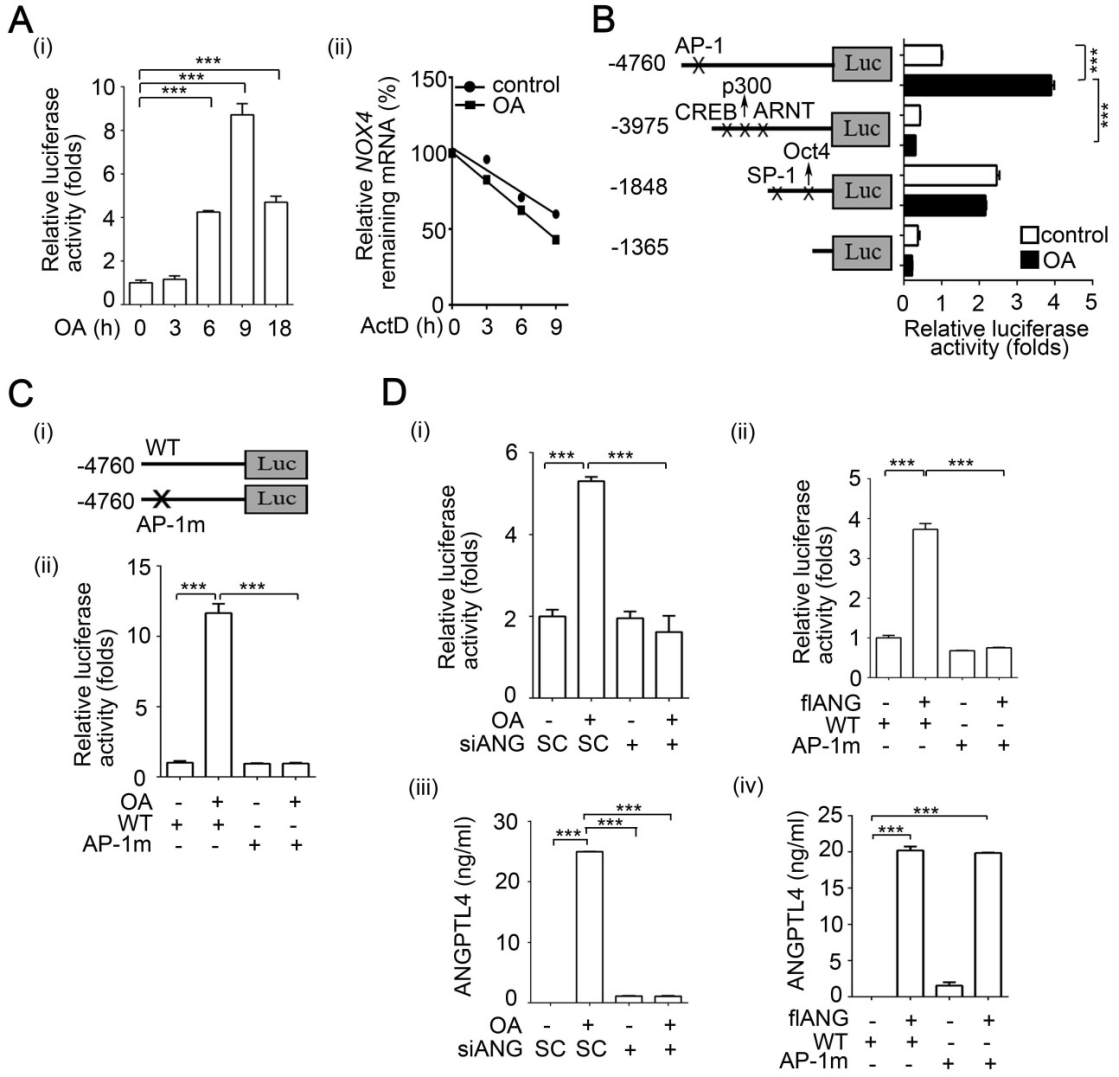
** OA-induced ANGPTL4 regulates NOX4 transcriptional activity through the AP-1 binding site on the NOX4 promoter. (A)** Dual-luciferase reporter assay was performed to analyze the activation of *NOX4* promoter (4.7 kb length) in SW480 cells. Cells were treated with 200 µM OA for the indicated period of time (i). The level of remaining *NOX4* mRNA was analyzed by real-time quantitative PCR in SW480 cells treated with or without 200 µM OA for 3 h followed by incubation with 4 μM actinomycin D (ActD) for the indicated period of time (ii). **(B)** Dual-luciferase reporter assay was performed in cells transfected with a series of 5'-truncated *NOX4* promoters followed by treatment with 200 µM OA for 16 h.** (C-D)** Dual-luciferase reporter assay and ELISAs were performed in SW480 cells transfected with the wild-type (WT) NOX4 promoter or with AP-1 binding site mutation (AP-1m) (C), and 20 nM siANGPTL4 (siANG) (D) (i, iii) or expression vector of ANGPTL4 (flANG) (D) (ii, iv), followed by treatment with 200 µM OA for 16 h. Firefly luciferase activity was determined and normalized to Renilla luciferase activity. The data are presented as the mean ± SEM. *P*-values were determined using a two-tailed Student's *t*-test. ***P < 0.001 (n=3).

**Figure 6:**
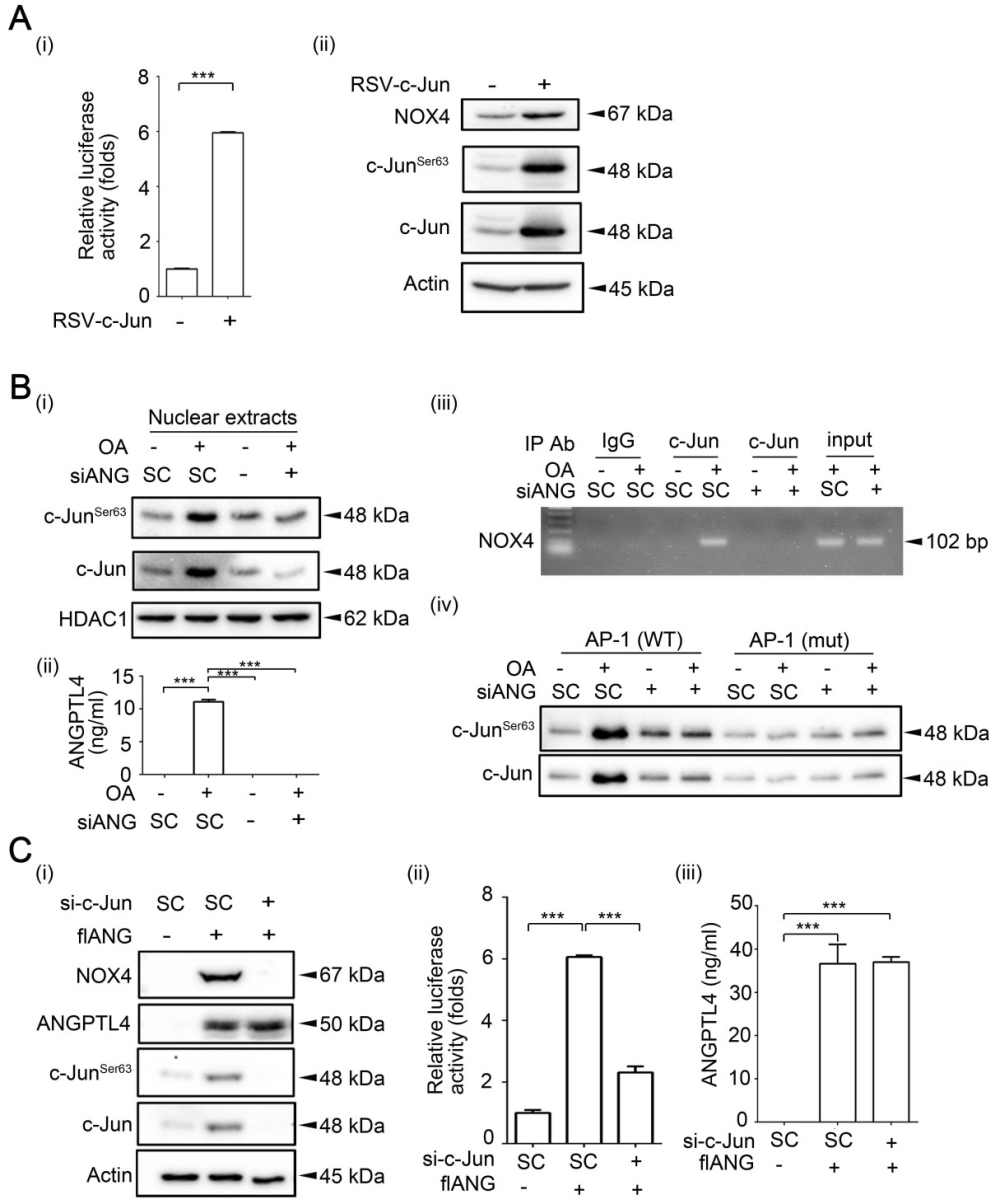
** c-Jun expression is essential for OA- and ANGPTL4-induced NOX4 transcriptional activity. (A)** Dual-luciferase reporter assay was performed to analyze the activation of *NOX4* promoter in SW480 cells transfected with the RSV-c-Jun expression vector for 24 h. Firefly luciferase activity was determined and normalized to Renilla luciferase activity (i). Immunoblot analysis of cell lysates form SW480 cells was performed using anti-phospho-c-Jun^Ser63^, anti-c-Jun, anti-NOX4, and anti-actin antibodies (ii). **(B)** Immunoblot analysis was performed using antibodies against HDAC1, c-Jun and phosphor-c-Jun^Ser63^ from SW480 cells transfected with 20 nM siANGPTL4 followed by treatment with 200 µM OA for 16 h (i). The secretion of ANGPTL4 was determined by ELISAs (ii). Chromatin immunoprecipitation (ChIP) assay (iii) and DNA affinity precipitation assay (iv) were performed to examine the binding of c-Jun to the *NOX4* promoter with wild-type (WT) and mutated (mut) AP-1 sites in SW480 cells transfected with 20 nM siANGPTL4 followed by treatment with 200 µM OA for 16 h as described in “Materials and methods”. **(C)** Protein levels of ANGPTL4, NOX4, c-Jun^Ser63^, c-Jun, and actin, NOX4 promoter activity, and the secretion of ANGPTL4 were determined by Immunoblot analysis (i), dual-luciferase reporter assay (ii) and ELISAs (iii) in cells transfected with 20 nM c-Jun siRNA (si-c-Jun) and expression vector of ANGPTL4 (flANG). The data are presented as the mean ± SEM. *P*-values determined using a two-tailed Student's *t*-test. ***P < 0.001 (n=3).

To clarify the correlation between ANGPTL4 and NOX4 in the regulation of OA-induced CRC cell invasion, the reciprocal effect of ANGPTL4 and NOX4 inhibition on ROS production was studied. As shown in Figure 4A and Figure S10, the depletion of ANGPTL4 inhibited fatty acid-induced ROS production in CRC cells. Treatment of cells with antioxidants inhibited ANGPTL4-induced ROS levels (Figure 4B). These results indicate the possibility that coincident expression of ANGPTL4 and NOX4 is required for OA-induced ROS production. Indeed, OA-induced NOX4 expression was inhibited in ANGPTL4 knockdown cells (Figure 4C and Figure S11). To further dissect the effect of ANGPTL4 isoforms on NOX4 expression, the full-length, C-terminal, and N-terminal ANGPTL4 expression vectors were used. As shown in Figure 4D, the full-length and C-terminal ANGPTL4, but not N-terminal ANGPTL4 dramatically induced NOX4 expression. However, there was no effect on OA-induced ANGPTL4 expression in NOX4-depleted or NAC-treated cells (Figure 4E). To study the molecular mechanism involved in regulation of OA-induced NOX4 expression, a reporter system was used for analysis of NOX4 promoter activity. As shown in Figure 5A, OA significantly induced NOX4 promoter activity in a time-dependent manner but without an effect on mRNA stability. The 5'-deletion constructs of the NOX4 gene promoter revealed that the AP-1 binding site between -4760 and -3975 bp of the promoter region was essential for the promoter activity responsible for the OA treatment effects (Figure 5B). The requirement of an AP-1 site for OA-induced NOX4 promoter activity was further confirmed in cells with an AP-1 mutant construct (Figure 5C). Furthermore, a depletion or increase in ANGPTL4 induced by siRNAs or expression vectors, respectively, suggested an essential role of ANGPTL4 expression in regulation of the transcriptional activity of the NOX4 gene (Figure 5D). On the other hand, overexpression of c-Jun, a component of AP-1, dramatically induced NOX4 promoter activity and protein expression (Figure 6A). The role of ANGPTL4 in the induction and activation of c-Jun to promote NOX4 gene expression was analyzed using DNA-protein binding assays including a chromatin immunoprecipitation (ChIP) assay and DNA affinity precipitation assay (DAPA). The results showed that the OA-induced expression and activation of c-Jun were inhibited in ANGPTL4 knockdown cells (Figure 6B). The OA-enhanced binding of c-Jun to the AP-1 binding site on the NOX4 promoter was also dramatically reduced by the depletion of ANGPTL4 as well as by the loss of binding between c-Jun and DNA, which was achieved with site-directed mutagenesis of the AP-1 binding site (Figure 6B). In addition, knockdown of c-Jun inhibited the ANGPTL4-induced NOX4 protein and promoter activities (Figure 6C). On the other hand, the inhibition of JNK pathway repressed the c-Jun phosphorylation and NOX4 expression, followed by the inhibition of ROS production, MMP-1 and MMP-9 expression, and invasion ability in OA-treated cancer cells (Figure S12). These results reveal that induction of ANGPTL4/c-Jun by OA is required for NOX4 expression which may contribute to ROS production and CRC cell invasion.

### NOX4 and ANGPTL4 expression is essential for OA-induced CRC extravasation and progression

**Figure 7:**
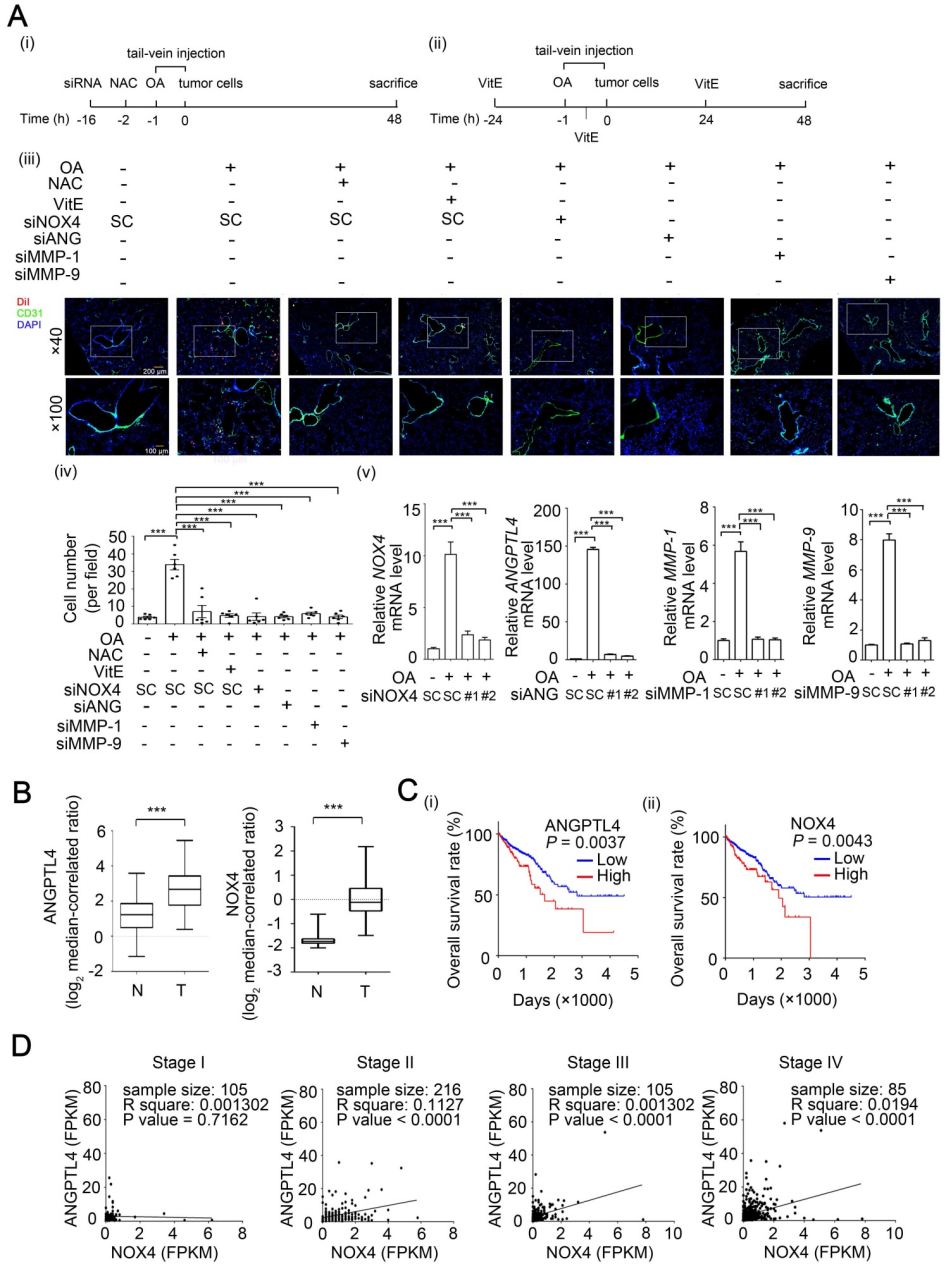
** The ANGPTL4/NOX4 axis is essential for OA-induced CRC extravasation and is associated with clinical outcome. (A)** Tumor cells penetrate to pulmonary blood vessels that was determined by *in vivo* extravasation assay. DiI staining of SW480 cells were transfected with 20 nM siNOX4, siANGPTL4, siMMP-1, and siMMP-9 or treated with 5 mM NAC and then injected intravenously into the tail vein of 6-week-old SCID-NOD mice which were preinjected intravenously with OA at a final concentration of 200 µM (i). The gavage feeding of vitamin E (VitE; 100 mg/kg body weight) is scheduled as indicated (ii). At 48 h after injection of tumor cells, the mice were sacrificed for examining of metastatic tumor cells surrounding the lung tissue as described in 'Materials and Methods'. Tumor cell penetration was imaged using a microscope (iii). Original magnification, × 40 and × 100; DiI labeled tumor cells (red); CD31 labeled blood vessels (green); DAPI labeled nucleus (blue). The number of tumor cell extravasation was calculated by analyzing at least four sections and six fields (iv); Six mice were analyzed for each group. Real-time quantitative PCR analysis was performed to determine *NOX4, ANGPTL4, MMP-1,* and* MMP-9* mRNA levels in SW480 cells treated with 200 µM OA for 48 h (v). Values are the mean ± SEM. ***P < 0.001, Student's *t*-test.** (B)** Box plots comparing the levels of *ANGPTL4* and *NOX4* mRNA in human CRC tissue (T) (n=65) and normal adjacent tissue (N) samples (n=65) were generated according to published data sets from Oncomine. ***P < 0.001, Student's *t*-test. Ref: Genes Chromosomes Cancer. 2010, 49:1024-34. **(C)** Kaplan-Meier curves showing CRC patient survival were retrieved from TCGA database. The median value was used to classify patients into high-expression or low-expression of ANGPTL4 (i) and NOX4 (ii). *P* values indicate the comparison between patients with high and low of ANGPTL4 or NOX4 expression. **(D)** Concurrent expression of ANGPTL4 and NOX4 in tumor tissues of CRC patients (n=597) in TCGA database was quantitated (Pearson's correlation coefficient is shown in the figures). FPKM: Fragments Per Kilobase of transcript per Million; Stages I~IV.

To further identify whether the ANGPTL4/NOX4 axis is required for OA-induced CRC metastasis, extravasation of CRC cells was analyzed using an *in vivo* mouse model. The mice were injected with OA through the tail vein to mimic hyperlipidemia conditions and then injected with CRC cells. Notably, the OA-pretreated mice presented an increase in tumor cells around the blood vessels of the surrounding lung tissues (Figure 7A). The depletion of NOX4, ANGPTL4, MMP-1, and MMP-9 significantly attenuated OA-induced CRC cell extravasation (Figure 7A and Figure S13A). Pretreatment of tumor cells with an ROS scavenger further suggested that the ROS production induced by hyperlipidemia was essential for tumor extravasation (Figure 7A). Intriguingly, hyperlipidemia-enhanced CRC extravasation was also inhibited in mice during gavage feeding of vitamin E (Figure 7A). Furthermore, depletion of NOX4, ANGPTL4, MMP-1, and MMP-9 in CRC cells significantly blocked OA-enhanced metastatic seeding of tumor cells in lungs (Figure S13B). In addition, the expression of NOX4 in tumors and secretion of ANGPTL4, MMP-1, and MMP-9 protein in surrounding lung tissues were observed in OA-primed parental but not siRNA knockdown cells (Figure S13B). On the other hand, higher NOX4 and ANGPTL4 expression was observed in tumors than in the surrounding normal tissues and was correlated with a lower survival rate of CRC patients (Figure 7B-C). The coincident expression of ANGPTL4 and NOX4 in tumors was highly associated with the late stage of CRC in patients (Figure 7D). Intriguingly, cytokines, including IL-6 and IL-8, are significantly increased in obese patients 35,36 and are used as markers that are correlated with hyperlipidemia. We found that IL-6 and IL-8 were induced by OA in CRC cells and associated with the expression of ANGPTL4 and NOX4 in CRC patients (Figure S14). These results reveal that the OA-induced ANGPTL4/NOX4 axis promotes CRC metastasis, suggesting that hyperlipidemia is associated with CRC progression.

## Discussion

**Figure 8:**
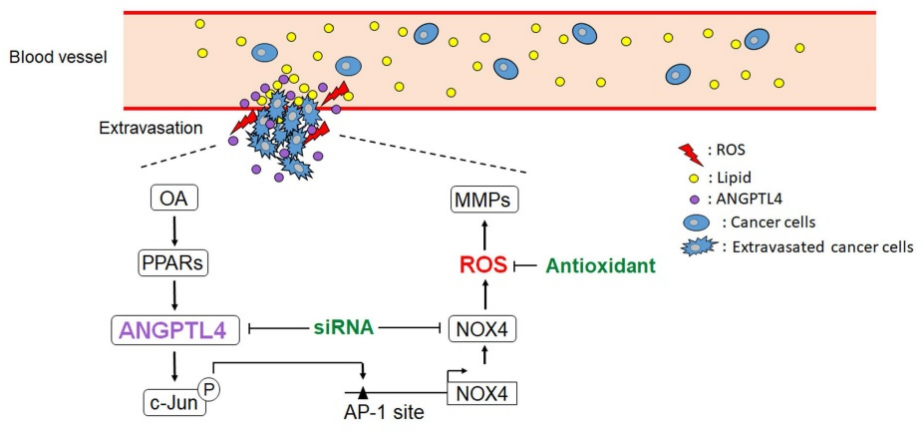
** Schematic diagram of the ANGPTL4/NOX4/ROS pathway in regulation of hyperlipidemia-induced CRC metastasis.** The metastatic properties of tumor cells are triggered by circulating lipids. OA-induced ANGPTL4 secretion further promotes NOX4 expression, which elevates ROS levels in cells, resulting in enhancement of tumor extravasation.

The dyslipidemia in metabolic syndrome is associated with various cancer types, such as breast, cervical, esophageal, and CRC 37-40. Although CRC progression has been linked with elevated circulating triglycerides, the mechanism involved in dyslipidemia-associated CRC metastasis remains unclear. For the first time, we found that OA induced NOX4 expression, resulting in an elevation of ROS levels that promoted CRC metastasis. The lipid loading imbalance, such as an increase in OA, triggered expression of the lipoprotein lipase inhibitor ANGPTL4 to prevent lipid overload in cells, followed by induction of NOX4 expression. These results suggest that the tumor cell response to hyperlipidemia through induction of the ANGPTL4/NOX4/ROS axis could be an underlying cause of CRC metastasis (Figure 8). Consistent with our findings, it has been found that either an HFD or hyperlipidemia is correlated with increased ROS levels and CRC metastasis 13,41-43. On the other hand, secretion of circulating ANGPTL4 in response to an elevated plasma ratio of FFAs further indicates the requirement of ANGPTL4 in regulation of hypertriglyceridemia 44. This evidence reveals that induction of ANGPTL4 lead to an increase in ROS levels, which promotes hyperlipidemia-associated CRC metastasis. In addition, concurrent ANGPTL4 and NOX4 expression was significantly present in patients with late-stage CRC, which suggests that a treatment that combines targeting of ANGPTL4 and the use of antioxidants could be considered for recurrent and metastatic CRC.

The effect of ROS levels on cancer is controversial; however, moderate ROS levels have been shown to support cancer drug resistance, proliferation, and metastasis 45,46. A recent report showed that metastatic cancer cells obtain an antioxidant capacity, resulting in an increased tolerance to higher ROS levels relative to primary tumor cells 47. However, use of antioxidants in clinical trials showed no significant benefit for cancer patients 48, possibly because antioxidants may not be able to target and reduce ROS generated and localized in the mitochondria 49,50. Therefore, the specific targeting of mitochondrial ROS or upstream ROS generators may enhance cancer therapeutic efficacy. Our results further suggest that inhibiting NOX4 by targeting fatty acid-induced ANGPTL4 may be a new therapeutic strategy. Indeed, ROS production can be modulated by fatty acids 51. Dietary fatty acids have been associated with oxidative stress and carcinogenesis in a rat model 52. Obese patients with dyslipidemia may present a loss of antioxidant capacity caused by low activity of the antioxidant enzyme SOD 53. These findings indicate that abnormal ROS production is associated with dyslipidemia, which is highly linked with tumor development. Although dyslipidemia has emerged as one of the leading environmental risk factors for CRC progression 54,55, the molecular mechanism involved in the regulation of FFA-associated CRC metastasis remains unknown. Here, we provide evidence to suggest that the induction of NOX4 by OA is at least one cause of CRC metastasis. Intriguingly, hyperlipidemia-enhanced tumor extravasation was reduced in mice treated with vitamin E. Despite the limitation of antioxidants in treating tumor development, we conclude that vitamin E could be considered to reduce metastatic cancer risk.

FFAs stimulate NOX expression in various cell types, including fibroblasts, endothelial cells, and monocytes 56-58. The NOX4 promoter harbors a hypoxia-responsive element that responds to hypoxia-inducible factor-1 during hypoxia in pulmonary artery smooth muscle cells 59. However, the mechanism involved in NOX4 expression stimulated by FFAs in CRC has not been investigated. Interestingly, we found that OA-induced NOX4 expression occurred through induction of ANGPTL4. In the physiology of obesity, increased FFAs stimulate ANGPTL4 secretion which inhibits LPL activity to reduce lipid loading. In addition, we found that the production of ANGPTL4 was stimulated by OA in CRC cells. These results suggest that secreted ANGPTL4 derived from stromal cells, adipocytes, or tumor cells may potentiate the expression of NOX4 to promote tumor metastasis. On the other hand, the ANGPTL4-regulated NOX4 gene expression was also dependent on the transcription factor c-Jun. These results are consistent with those of our previous studies indicating that activation of c-Jun is required for ANGPTL4-regulated gene expression, such as MMP-1 expression 60. Therefore, the regulation of ROS production in the presence of increased FFA levels may be caused by ANGPTL4 secretion, which leads to NOX4 expression and cancer metastasis.

Despite the controversial role of NOX4 in tumor progression, including the enhancement of prostate cancer cell progression but inhibition of liver cancer cell proliferation 18,20, we found that increased NOX4 levels were associated with the poor survival rates of CRC patients and with tumor metastasis. Several previous studies also support the oncogenic effects of NOX4 in tumors. For example, hypoxia-induced IL-6 and IL-8 promote renal cell carcinoma cell invasion, which is dependent on NOX4 expression 61. The effect of NOX-enhanced tumor metastasis in gastric cancer could be mediated through regulation of EMT markers, including MMPs, fibronectin, vimentin, snail, zeb1, and E-cadherin 62, which is consistent with our findings that MMP expression was essential for NOX4-regulated CRC cell invasion. These results reveal that the expression of NOX4 is highly correlated with cancer metastasis. NOX4-mediated cancer metastasis was further confirmed in our studies by the depletion of NOX4, which significantly inhibited OA-induced extravasation and tumor nodule formation in the lungs of mice.

ANGPTL4 has been identified as an adipokine that regulates lipid metabolism and plays roles in cancer progression; however, there is still some controversy regarding its participation in cancer metastasis. Early studies proposed an anti-angiogenic role for ANGPTL4; therefore, ANGPTL4 could prevent metastasis by inhibiting vascular leakiness 63. ANGPTL4 was also reported to prevent metastasis through inhibition of angiogenesis, tumor cell motility and invasion 64. On the other hand, several reports also suggest that ANGPTL4 enhances tumor metastasis by triggering endothelial disruption or alteration of MMP expression in tumor cells 65. These conflicting results indicate that ANGPTL4 acts as a tumor suppressor or promoter in cancer metastasis, depending on the cell type and stage of the cancer 66. However, our present study together with our previous study 60 provide evidence suggesting that ANGPTL4 has the ability to promote CRC and HNSCC metastasis via modulation of ROS levels and MMP activity. In addition, activation of pathways downstream of integrins, including ERK and PI3K, is required for ANGPTL4-promoted hepatocellular carcinoma metastasis 28. Our previous studies also suggest that integrin β1 signaling pathway was at least partially essential for ANGPTL4-induced MMP-1 expression 60. These results reveal that ANGPTL4-triggered tumor metastasis could occur through activation of integrin signaling. Therefore, further clarification of whether activation of integrin signaling pathways is essential for OA-induced NOX4 expression would be interesting.

Although we found that NOX4 and ANGPTL4 expression was associated with CRC progression in a clinical database, the coincidence of CRC metastasis and hyperlipidemia in patients should be further examined. Notably, an increase in cytokine production, such as IL-6 and IL-8, was accompanied by higher levels of fatty acids in patients with ulcerative colitis 67. The EMT process can be triggered by IL-6 and IL-8 in breast cancer and CRC cells 68,69. This suggests that hyperlipidemia-associated CRC metastasis may be caused by production of cytokines, such as IL-6 and IL-8. Indeed, we also found that IL-6 and IL-8 expression was increased in CRC patients, as shown in the clinical database, and in OA-treated tumor cells. These results indicate that IL-6 and IL-8 production may link hyperlipidemia with CRC metastasis. Further dissection of whether ANGPTL4 expression is regulated by hyperlipidemia-induced cytokines would be of interest.

By identifying that the induction of ANGPTL4 results in an increased ROS production level, the results of this study suggest a potential mechanism for hyperlipidemia-regulated CRC metastasis and indicate that ANGPTL4 may be a potential target for elimination or prevention of CRC metastasis. Thus, this study provides a new understanding of how the dyslipidemia-induced ANGPTL4/NOX4 axis contributes to CRC metastasis. However, whether a combination of antibodies targeting ANGPTL4 and antioxidants can lead to reduction of CRC recurrence is still unknown. Further investigations are warranted to investigate whether ANGPTL4 and/or NOX4 represent a viable target for clinical treatment, particularly for hyperlipidemia patients with metastatic CRC.

## Supplementary Material

Supplementary figures and tables.
