# Supplementary material for: Oleic acid-induced NOX4 is dependent on ANGPTL4 expression to promote human colorectal cancer metastasis
Source: Theranostics. 2020 May 30;10(16):7083–99. doi: 10.7150/thno.44744 (PMC7330862; doi:10.7150/thno.44744)
Supplement: Supplementary file 1 — Supplementary figures and tables. [file thnov10p7083s1.pdf]

Oleic acid-induced NOX4 is dependent on ANGPTL4 expression to promote human colorectal cancer metastasis

Shen *et al*.

Supplementary Materials and Methods

Supplementary Figures

Supplementary materials and methods

Reagents and peptide

Salt oleic acid (sodium oleate), Linoleic acid sodium salt, Sodium palmitate, actinomycin D, N-acetylcysteine (NAC), (+)-α-Tocopherol (T1539), and Mito-TEMPO were purchased from Sigma-Aldrich (Sigma-Aldrich, St Louis, MO, USA). Bovine serum albumin (fatty acid free) (US Biological, Swampscott, MA, USA) was used to be conjugated with oleic acid with 1:8 molar ratios (BSA/OA). Carboxyl H2-DCFDA, CFSE, and mitoSOX was purchased from Invitrogene (Invitrogen, Grand Island, NY, USA). Recombinant human ANGPTL4 (rh-ANGPTL4) was purchased from R&D Systems (R&D Systems, Minneapolis, MN). PPAR antagonists, GW9662, GSK0660, and GW6471 were purchased from Sigma-Aldrich.

Enzyme-linked immunosorbent assay

Quantification of the secretory ANGPTL4 in the culture medium was achieved by a DuoSet® Human Angiopoietin-like 4 Enzyme-linked immunosorbent assay (ELISA) development kit (DY3458) according to the manufacturer's instruction (R&D systems). Briefly, 100 μl of culture medium was collected and incubated with pre-coated capture antibody at room temperature in a 96-well microplate. After washing step, 100 μl of detection antibody was added and incubated for another 2 h at room temperature, and then add 100 μl of the working dilution of Streptavidin-HRP to each well and incubated for 20 min under protecting from light. After washing step, 100 μl of substrate solution was added and incubated for 20 min under protecting from light. After adding 50 μl of stop solution, gently tapped the plate to ensure thorough mixing and used an SpectraMax i3x Microplate Absorbance Reader (Molecular Devices, CA, USA) to quantify the optical density at a wavelength of 450 nm immediately.

Luciferase assays

For determination of luciferase activity by using with the pGL3 basic Luciferase Reporter Vectors system (Promega, Madison, WI, USA), the transfected cells (3×10^5) were washed with phosphate-buffered saline (PBS) and lysed in 150 μl of Cell Culture Lysis Reagent (CCLR; Promega) and stand at room temperature for 15 min. The transfection efficiency was estimated by co-transfected with Renilla plasmid and the Renilla luciferase activity was performed by using Renilla Luciferase Assay System (Promega). The cell lysate was centrifuged at $7200 \times g$ for 30 secs, and 30 μl of the supernatant solution was mixed with luciferase assay substrates according to the manufacturer's protocol. The luciferase activity was measured by a MiniLumat LB 9506 luminometer (EG&G Berthold, Bad Wildbad, Germany) and normalized to Renilla luciferase activity.

Zymography assay

Conditioned medium was electrophoresed in a 8% polyacrylamide gel containing 0.1% (w/v) Type I collagen (Corning Inc., Corning, NY, USA). The gel was then washed at room temperature for 30 min with 2.5% Triton X-100 and subsequently incubated at 37°C for 24 h in a buffer containing 10 mM $CaCl_2$, 0.01% NaN3, and 50 mM Tris–HCl (pH 7.5). The gel was stained with 0.2% Coomassie brilliant blue and photographed on a light box. Proteolysis was detected as a white zone in a dark blue field. The intensity was quantified with ImageJ software (http://rsb.info.nih.gov/ij/).

Lentivirus knockdown assay

RNA interference vectors used in this study were obtained from the National RNAi Core Facility in the Institute of Molecular Biology, Academia Sinica (Taipei, TWN).

The lentivirus of hairpins targeting PPARα (shPPARα), PPARγ (shPPARγ), PPARδ (shPPARδ), and scramble (shSC) were packaged and obtained from RNAi Core of National Cheng Kung University Hospital in the pLKO.1 lentiviral backbone. The accession numbers in the RNAi core were as follows: shPPARα: TRCN0000001665, shPPARγ: TRCN0000001673, shPPARδ: TRCN0000001664, shSC: ASN0000000001. Briefly, 1×10^5 cells were seeded to each well in 6-well plates for overnight. Before infection of lentivirus, the culture media was changed to the fresh media containing 8 μg/ml polybrene (Sigma-Aldrich). Lentivirus was added to the cells at MOI = 3. Following additional incubation of infected cells overnight, infected cells was selected with 2 μg/ml puromycin (Sigma-Aldrich) for 1 week and then maintained with 1 μg/ml puromycin before using for experiments.

Reverse transcription polymerase chain reaction (RT-PCR) and primer sets

Total RNA of cells was extracted from cells by using TRIzol® (Invitrogen, Carlsbad, CA, USA). A reverse transcriptase reaction was performed using reverse transcriptase reaction kit (Applied Biosystems, Foster City, CA, USA) according to the manufacturer's instructions. Primer sets for RT-PCR or real-time quantitative PCR were the following: NOX1 specific primers (sense, 5’- CGC TCC CAG CAG AAG GTT GTG ATT ACC AAG G -3’; antisense, 5’- GGG GTG ACC CCA ATT CCT GCT CCA ACC A -3’), NOX2 specific primers (sense, 5’- ACT TGC AGG CTT TGT ATG TGA A -3’; antisense, 5’- ACA GTT TTA AGA ATT CCC CGT AGA -3’), NOX3 specific primers (sense, 5’- GAC TTC TGG CCG CAC TTT C -3’; antisense, 5’- CAG GGT TGA GGT AGC TCT CG -3’), NOX4 specific primers (sense, 5’- CTC AGC GGA ATC AAT CAG CTG TG -3’; antisense, 5’- AGA GGA ACA CGA CAA TCA GCC TTA G -3’), NOX5 specific primers (sense, 5’- GCT TAT CCG AGG TCT CAA TCC -3’; antisense, 5’- AAG AAC CCA CCA ATT CCA GA -3’), SOD1 specific

primers (sense, 5’- GCA TCA TCA ATT TCG AGC AG -3’; antisense, 5’- ACA GCC TGC TGT ATT ATC TC -3’), SOD2 specific primers (sense, 5’- GAA CAA CAG GCC TTA TTC CAC -3’; antisense, 5’- TGC TAC AAT AGA GCA GCT TAC -3’), MMP-1 specific primers (sense, 5’- ATG CAC AGC TTT CCT CCA CT -3’; antisense, 5’- TTC CCA GTC ACT TTC AGC CC -3’), MMP-3 specific primers (sense, 5’- GCA AGA CAG CAA GGC ATA GAG -3’; antisense, 5’- CCG TCA CCT CCA ATC CAA GG -3’), MMP-9 specific primers (sense, 5’- ACC TCG AAC TTT GAC AGC GAC A -3’; antisense, 5’- GAT GCC ATT CAC GTC GTC CTT A -3’), Vimentin specific primers (sense, 5’- TGG CCG ACG CCA TCA ACA CC -3’; antisense, 5’- CAC CTC GAC GCG GGC TTT GT -3’), E-cadherin specific primers (sense, 5’- TCC ATT TCT TGG TCT ACG CC -3’; antisense, 5’- CAC CTT CAG CCA ACC TGT TT -3’), Zeb-1 specific primers (sense, 5’- GCC AAT AAG CAA ACG ATT CTG -3’; antisense, 5’- TTT GGC TGG ATC ACT TTC AAG -3’), PPAR α specific primers (sense, 5’- CAG AAC AAG GAG GCG GAG GTC -3’; antisense, 5’- TTC AGG TCC AAG TTT GCG AAG C -3’), PPAR γ specific primers (sense, 5’- GAG CCC AAG TTT GAG TTT GC -3’; antisense, 5’- CAG GGC TTG TAG CAG GTT GT -3’), PPAR δ specific primers (sense, 5’- TGG CTT TGT CAC CCG TGA GT -3’; antisense, 5’- ACA GAA TGA TGG CCG CAA TGA A -3’), IL-8 specific primers (sense, 5’- TTG GCA GCC TTC CTG ATT TCT -3’; antisense, 5’- TCT CAG CCC TCT TCA AAA ACT TCT C -3’), IL-6 specific primers (sense, 5’- ATG AAC TCC TTC TCC ACA AGC -3’; antisense, 5’- GTT TTC TGC CAG TGC CTC TTT G -3’), ANGPTL4 specific primers (sense, 5’- CAA GGC TCA GAA CAG CAG GA -3’; antisense, 5’- CCC CTG AGG CTG GAT TTC AA -3’), Duox1 specific primers (sense, 5’- CAC CTC CTG GAG ACC TTT TTC -3’; antisense, 5’- GGC CTG GTT GAT GTC CAG -3’). Duox2 specific primers (sense, 5’- TAC CCA GGA GGT GAA TGA GC -3’; antisense, 5’- TCC TCC AAC TCC GAA ATC TG -3’). PTEN specific primers (sense, 5’- CAA GAT GAT

GTT TGA AAC TAT TCC AAT G -3'; antisense, 5'- CCT TTA GCT GGC AGA CCA CAA -3'). ACOX1a specific primers (sense, 5'- TGC TCA GAA AGA GAA ATG GC -3'; antisense, 5'- TGG GTT TCA GGG TCA TAC G -3'). GAPDH specific primers (sense, 5'- CCA TCA CCA TCT TCC AGG AG -3'; antisense, 5'- CCT GCT TCA CCA CCT TCT TG -3'). The PCR products were separated by 2% agarose gel electrophoresis and visualized with ethidium bromide (Sigma-Aldrich) staining.

Real-time quantitative PCR

Following cDNA synthesis, gene-specific primers were designed using NCBI primer design software. Real-time quantitative PCR was performed in triplicate using a SYBER Green MasterMix (Invitrogen) and an StepOne Real-Time PCR Systems (Applied Biosystems, Carlsbad, CA, USA). Each well contained the following reaction mixture: 1 μl of cDNA, 5 μl of 2 × SensiMix Syber Green PCR reagents (Bioline, London, UK), 4 μl of RNase-free water, 0.5 μl of sense primer, and 0.5 μl of antisense primer. Universal cycling conditions were used. Relative gene expression was calculated using the comparative Ct method. All values were normalized to the housekeeping gene GAPDH.

Western blotting

An analytical 12% SDS-PAGE was performed, and 30 μg of protein of each were analyzed. Western blotting was performed as previously described [1]. Antibodies against human NOX4 (GTX121929) and MMP-9 (GTX100458) (GeneTex, Hsinchu, TWN), ANGPTL4 (#409800) (Invitrogene), c-Jun (#61327) (BD Tranduction Laboratory, Los Angeles, CA, USA), IκBα (#9242), NF-κB p65 (#8242), phospho-c-Jun^{Ser63} (#2361) (Cell Signaling Technology, Inc., Danvers, MA, USA), Actin (A5441) (Sigma-Aldrich), GAPDH (sc-32233), MMP-1 (sc-21731), HDAC1 (sc-8410), JNK1

(sc-474), and p-JNK (sc6254) (Santa Cruz Biotechnology, Inc, Santa Cruz, CA, USA) were used as the primary antibodies.

CFSE proliferation assay

Cells were harvested and resuspended in 1 ml medium. Cells were labelled by the addition of 1 ml of PBS containing 5 μM the carboxyfluorescein succinimidyl ester (CFSE), followed by 5 min incubation at room temperature. Labelled cells were then washed twice, counted and seeded at 1×10^5 cells/well in a 12 well plate. On the time of analysis for 24, 48, 72 h, cells were harvested and washed twice with PBS and then CFSE intensity was examined by FACS. Experiments were performed at least three times and data presented are of one representative experiment.

Quantitative estimation of H_2O_2 concentration

The quantitative estimation of H_2O_2 was carried out using a Hydrogen Peroxide Assay kit (Cat. No. K265-200) purchased from BioVision (BioCat, Heidelberg, Germany). Cells was lysis in 0.5 mL of PBS containing 1% Triton X-100. Cell lysates was then at 10,000 g at 4°C for 30 min and supernatant was used immediately for quantitative estimation of H_2O_2. The optical density (OD) was determined using a microplate reader set to 550 nm. Samples from three independent experiments were used for each group and all samples were run in one assay to avoid inter-assay variation.

RNA stability assay

To determine the half-life of OA-induced mRNA expression of gene, cells were treated with OA for 3 h, and then the 5 μg/ml Actinomycin D (Sigma) was added into the growth medium to block transcription. During the following 3, 6 ,9 h, cells were harvested and total RNA was prepared by TRIzol® (Invitrogen Life Technologies).

cDNA was prepared by reverse transcriptase reaction by using reverse transcriptase reaction kit (Invitrogen Life Technologies) according to the manufacturer's instructions and the expression of target genes were analyzed by real-time quantitative PCR.

DNA affinity precipitation assay

Quantitation of the change of c-Jun binding to the NOX4 promoter element was achieved by a DNA affinity precipitation assay (DAPA) according to the method as previously described [2]. In brief, 5’-biotinylated oligonucleotides and corresponding to the sense -4679 to -4650 bp and antisense strands of the NOX4 promoter element were annealed. The DAPA was performed by incubating 2 μg of biotinylated DNA probe listed as follows: WT NOX4 annealing probe (sense, 5’- TAG AAA TGA GAA TGA GTC ACT GTC TGG AAC -3’; antisense, 5’- GTT CCA GAC AGT GAC TCA TTC TCA TTT CTA -3’); AP-1m NOX4 annealing probe (sense, 5’- TAG AAA TGA GAA TGA GT**T G**CT GTC TGG AAC -3’; antisense, 5’- GTT CCA GAC AG**C** **A**AC TCA TTC TCA TTT CTA -3’). Mutated positions in the sequence of the primers were underlined. Probes were then mixed with 200 μg of nuclear extract and 20 μl of streptavidin agarose beads in PBS at room temperature for 1 h with rotation. Beads were collected and washed three times with cold PBS. The binding proteins were eluted by loading buffer and separated by SDS-PAGE, followed by Western blot analysis probed with specific antibodies against c-Jun^{Ser63} or c-Jun.

Plasmid construction

Truncated lengths of NOX4 promoter were constructed by primer sets in the previous study [3]. Especially, NOX4 promoter (1.84 kb) was amplified with primer sets: sense, 5’- GGA CGC GTG GCT CAC CGC AAC CTC TGC CTC -3’; antisense, 5’- GGC TCG AGG CTG CCC AGA CGC CCA GCG C -3’, and cloned into PGL3 basic vector

(Promega) by using *Mlu*I and *Xho*I restriction enzyme (NEB Biolabs). The mutant was constructed by the site-directed mutagenesis method with Phusion® Hot start Flex DNA polymerase (NEB Biolabs). Synthetic primers of site-directed mutagenesis were listed as follows: pNOX4-AP-1M sense primer 5'- **G**TA GAA ATG AGA ATG AGT **TG**C TGT CTG GAA CTT GTC -3'; pNOX4-AP-1M antisense primer 5'- GAC AAG TTC CAG ACA G**CA** ACT CAT TCT CAT TTC TAC -3. Mutated positions in the sequence of the primers were underlined. The vector sequence was confirmed by DNA sequencing. The expression vectors of full-length (flANGPTL4), C-terminal (cANGPTL4), and N-terminal (nANGPTL4) ANGPTL4 and ANGPTL4-PPRE promoter vector were constructed as previously described [4]. NOX4 overexpression vector was purchased from Sino Biological Inc. (Beijing, CN). Dominant negative IκB (DN- IκB) mutant was generated by N-terminal deletion of residues 1-45 using a standard PCR approach [5].

Tumor metastasis assay in an animal model

Tumor metastasis was determined by tail vein intravenous injection of cancer cells into 6- to 8-week-old male severe combined immunodeficiency mice. There is no randomization method and blinding group for allocation of animals. Briefly, 5×10^5 tumor cells were resuspended in 100 μl with Hank's Balanced Salt Solution and then injected into the tail vein of mice. To evaluate lung metastasis, mice were sacrificed up to 1.5 months after injection. H&E staining was performed by the Human Biobank, Research Center of Clinical Medicine, NCKU Hospital. Quantification was performed by analysing whole lung to determine the number of nodules that underwent metastasis. All mice were obtained from the National Cheng Kung University (NCKU) Laboratory Animal Center (Tainan, TWN). The animal study was approved (Approved No.

NCKU-IACUC-107-112) by the IACUC of Laboratory Animal Center, Medical College, NCKU.

Immunohistochemistry assay

Immunohistochemistry (IHC) was performed essentially as previously described [6]. Briefly, endogenous peroxidase activity of tissue sections was blocked with H_2O_2. Slides were incubated in boiling citrate buffer (pH 6.0), then maintained at a sub-boiling temperature for 10 min. Cool slides on bench top for 30 min. The sections were incubated at 4°C for overnight with anti-NOX4 (diluted 1:100) (ab109225; Abcam). anti-ANGPTL4 (diluted 1:100) (A00309-02-100; Aviscera), anti-MMP-1 (diluted 1:100) (sc-58377; Santa Cruz), anti-MMP-9 (diluted 1:100) (GTX100458; GeneTex) antibodies. The reaction complexes were detected using a kit (VECTASTAIN Elite ABC kits) and visualized using a 3, 3-diaminobenzidine (DAB) substrate kit (Vector DAB substrate). The image of immunohistochemistry was taken by a microscopy.

Figure legends

Figure S1. OA-induced ROS levels are partially produced from mitochondrial in SW480 cells. (A-B) ROS levels were examined by flow cytometry analysis with DCFDA (A and B (i)) or MitoSOX (B (ii)) staining in cells treated with 200 μM OA, H_2O_2, and Mito-TEMPO for 24 h. Quantification of ROS intensities is shown in columns. BG indicates background. The data are presented as the mean ± SEM. *P*-values were determined using a two-tailed Student's *t*-test. ***$P < 0.001$ (n=3).

Figure S2. Fatty acids-induced NOX4 expression regulates ROS production in colorectal cancer cell lines. (A-C) The expression of NOX4 was determined by immunoblotting using anti-NOX4 antibodies in CRC cells-treated with 200 μM OA for 16 h (A), treated with 200 μM OA as indicated time (B), or SW480 cells treated with 200 μM LA and PA for 16 h (C). (D-E) ROS levels and NOX4 mRNA expression were estimated by flow cytometry analysis with DCFDA staining and real-time quantitative PCR, respectively in SW480 cells transfected with 20 nM NOX4 siRNA and then treated with 200 μM LA and PA for 16 h. The quantification of ROS intensities (i) and mRNA levels (ii) is shown in columns. BG indicates background. The data are presented as the mean ± SEM. *P*-values were determined using a two-tailed Student's *t*-test. ***$P < 0.001$ (n=3).

Figure S3. OA-induced NOX4 regulates H_2O_2 levels in colorectal cancer cell lines. (A) The hydrogen peroxide assay kit was used to determine H_2O_2 levels in cell lysates, and real-time quantitative PCR was performed to assess the NOX4 mRNA level in SW480 (i) and HT-29 (ii) cells transfected with 20 nM NOX4 siRNA and then treated with 200 μM OA for 16 h. The data are presented as the mean ± SEM. *P*-values were determined using a two-tailed Student's *t*-test. ***$P < 0.001$ (n=3). (B) ROS levels

were estimated by flow cytometry analysis with DCFDA staining (i) and immunoblot analysis was performed using anti-NOX4 and anti-GAPDH antibodies (ii) in NOX4 overexpressing SW480 cells treated with or without 5 mM NAC for 24 h. BG indicates background. Vec indicates empty vector. The data are presented as the mean ± SEM. *P*-values were determined using a two-tailed Student's *t*-test. ***$P < 0.001$ (n=3).

Figure S4. Fatty acids-induced invasion ability and effects of an antioxidant on ROS production, and MMPs expression in SW480 cells. (A-B) Invasion assays were performed with 100 or 200 μM OA-, 200 μM LA-, and 200 μM PA-treated cells for 72 h. Crystal violet-stained invading cells were imaged with a microscope (i), and then solubilized in 10% acetic acid. The absorbance was measured at a wavelength of 595 nm (ii). The data are presented as the mean ± SEM. (C) ROS levels were estimated by flow cytometry analysis with DCFDA staining in cells treated with 200 μM OA and NAC (i) or vitamin E (VitE) (ii) for 24 h. Quantification of ROS intensities is shown in columns. BG indicates background. (D) Semi- and real-time quantitative PCR analysis of *MMP-1*, *MMP-3*, and *MMP-9* mRNA levels were performed in cells treated with 200 μM OA and/or 5 mM NAC and 15 μM VitE for 24 h. The data are presented as the mean ± SEM. *P*-values were determined using a two-tailed Student's *t*-test. ***$P <$ 0.001 (n=3).

Figure S5. Expressions of NOX4 and MMP-3 are essential for OA-induced invasion ability. (A and E) An invasion assay was performed in HT-29 (A) and SW480 (E) cells transfected with siNOX4 and siMMP-3 siRNA, respectively and then treated with 200 μM OA for 72 h. Crystal violet-stained invading cells were imaged with a microscope (i), and then solubilized in 10% acetic acid. The absorbance was measured at a wavelength of 595 nm (ii). Expressions of NOX4 and MMP-3 mRNA were

determined by real-time quantitative PCR (iii). (B) Expressions of MMP-1, MMP-3, MMP-9, and NOX4 were examined using real-time quantitative PCR analysis in HT-29 cells transfected with 20 nM NOX4 siRNA, and then treated with 200 μM OA for 16 h. (C) Expressions of MMP-1 and MMP-9 were estimated by immunoblotting in SW480 cells transfected with 20 nM scrambled oligonucleotides (SC), MMP-1, and MMP-9 siRNA for 24 h and then treated with 200 μM OA for 24 h. (D) Zymography assay was performed to assess MMP-3 activity in SW480 cells transfected with 20 nM NOX4 and ANGPTL4 siRNA and treated with 200 μM OA for 16 h or transfected with pCMV-NOX4 and treated with 5 mM NAC and 15 μM vitamin E (VitE). Vec indicates empty vector. The data are presented as the mean ± SEM. *P*-values were determined using a two-tailed Student's *t*-test. ***$P < 0.001$ (n=3).

Figure S6. The proliferation of SW480 cells is not regulated by OA, NOX4, ANGPTL4, MMP-1, and MMP-9. (A-B) Proliferation assays were performed through CFSE staining and quantification by flow cytometry in cells transfected with 20 nM siRNA targeting NOX4, ANGPTL4, MMP-1, and MMP-9 genes and then treated with 200 μM OA for period of time as indicated (A) or with OA for 72 h (B). CFSE emission was measured at a wavelength of 525 nm (FL1). BG indicates background.

Figure S7. An antioxidant and depletion of MMP-1 and MMP-9 reduce NOX4-induced invasion ability in SW480 cells. (A-B) Invasion ability was examined in cells transfected with pCMV-NOX4 and then treated with 5 mM NAC for 72 h (A), or co-transfected with MMP-1 or MMP-9 siRNA (B). Crystal violet-stained invading cells were imaged with a microscope (i), and then solubilized in 10% acetic acid. The absorbance was measured at a wavelength of 595 nm (ii). Real-time quantitative PCR analysis of *NOX4, MMP-1, MMP-3, MMP-9, Vimentin, E-cadherin, and ZEB1* mRNA

levels was performed (iii). The expression of NOX4 protein was determined by immunoblotting (iv). Vec indicates empty vector. The data are presented as the mean ± SEM. *P*-values were determined using a two-tailed Student's *t*-test. ***$P < 0.001$ (n=3). (C) Immunoblotting was performed using antibodies against MMP-1, MMP-9, NOX4, NF-κB p65, IκBα, and GAPDH in cells transfected with pCMV-NOX4 and dominant negative IκB (DN-IκB) expression vector.

Figure S8. The expression of PPARδ is essential for OA-induced ANGPTL4 expression. (A-B) OA-regulated ANGPTL4 expression was examined using semi- (A) (i) and real-time (A) (ii) quantitative PCR analysis, and dual-luciferase reporter assay (B). CRC cells (i) and HT-29 cells (ii) were treated with 200 μM OA for 24 h or periods of time as indicated, respectively. SW480 cells were transfected with *ANGPTL4* promoter with PPAR response element (PPRE) and followed by treatment with 200 μM OA for 24 h. (C-D) ANGPTL4 promoter activity and PPARs gene expression were examined using dual-luciferase reporter assay (C) and semi-time quantitative PCR analysis (D), respectively. SW480 cells were treated with various concentrations or periods of time of OA as indicated. (E) The real-time quantitative PCR analysis (i) and dual luciferase reporter assay (ii) were used to examine the *ANGPTL4*, *PTEN*, and *ACOX1a* mRNA levels and ANGPTL4 promoter activity, respectively. SW480 cells were transfected with ANGPTL4 promoter followed by treatment with inhibitors of 5 μM PPARδ (GSK0660), 1 μM PPARα (GW6471), and 5 μM PPARγ (GW9662), and 200 μM OA for 24 h. Firefly luciferase activity was determined and normalized to Renilla luciferase activity. The induction folds were normalized to control value. The data are presented as the mean ± SEM. *P*-values were determined using a two-tailed Student's *t*-test. *$P < 0.05$; **$P < 0.01$; ***$P < 0.001$ (n=3).

Figure S9. The Expression of ANGPTL4 is essential for OA-induced invasion ability of HT-29 cells. (A) An invasion assay was performed in cells transfected with 20 nM ANGPTL4 siRNA and then treated with 200 μM OA for 72 h. Crystal violet-stained invading cells were imaged with a microscope (i), and then solubilized in 10% acetic acid. The absorbance was measured at a wavelength of 595 nm (ii). Expressions of *ANGPTL4* mRNA were determined by real-time quantitative PCR (iii). (B) Expressions of MMP-1, MMP-3, MMP-9, and ANGPTL4 were examined using real-time quantitative PCR analysis in cells transfected with 20 nM ANGPTL4 siRNA, and then treated with 200 μM OA for 16 h. The data are presented as the mean ± SEM. *P*-values were determined using a two-tailed Student's *t*-test. ***$P < 0.001$ (n=3).

Figure S10. Fatty acids induced-ANGPTL4 stimulates ROS production in CRC cells. (A-C) ROS levels were estimated by flow cytometry analysis with DCFDA staining in HT29 (A) and SW480 (B-C**)** cells transfected with 20 nM ANGPTL4 siRNA and then treated with 200 μM OA (A), LA (B) and PA (C) for 24 h. Quantification of ROS intensities is shown in columns (i). Real-time quantitative PCR analysis was used to examine *ANGPTL4* mRNA level in cells treated with 200 μM fatty acids for 16 h (ii). The data are presented as the mean ± SEM. *P*-values were determined using a two-tailed Student's *t*-test. ***$P < 0.001$ (n=3).

Figure S11. The depletion of ANGPTL4 reduces OA-induced NOX4 expression in HT-29 cells. Real-time quantitative PCR analysis of *ANGPTL4* and *NOX4* mRNA levels was performed in cells transfected with 20 nM ANGPTL4 siRNA and SC siRNA and then treated with 200 μM OA for 16 h. The data are presented as the mean ± SEM. *P*-values were determined using a two-tailed Student's *t*-test. ***$P < 0.001$ (n=3).

Figure S12. OA-induced c-Jun activation, expressions of NOX4 and MMPs, ROS production, and invasion ability are inhibited in JNK inhibitor-treated SW480 cells. (A-B) The expression of proteins including NOX4, JNK, phospho-c-Jun (p-c-Jun), phosphor-JNK (p-JNK), and GAPDH, and mRNA levels including *NOX4*, *MMP-1,* and *MMP-9* were determined by immunoblotting (A) and real-time quantitative PCR (B), respectively. SW480 cells were treated with 200 μM OA and/ or 20 μM SP600125 for 16 h. (C) ROS levels were estimated by flow cytometry analysis with DCFDA staining in SW480 cells treated with 200 μM OA and/ or 20 μM SP600125 for 24 h. Quantification of ROS intensities is shown in columns. (D) An invasion assay was performed in SW480 cells transfected with 200 μM OA and/ or 20 μM SP600125 for 72 h. Crystal violet-stained invading cells were imaged with a microscope (i), and then solubilized in 10% acetic acid. The absorbance was measured at a wavelength of 595 nm (ii). The data are presented as the mean ± SEM. *P*-values were determined using a two-tailed Student's *t*-test. ***$P < 0.001$ (n=3).

Figure S13. The ANGPTL4/NOX4/MMP axis is essential for OA-induced metastasis in CRC cells. (A-B) The penetration of tumor cells to pulmonary blood vessels and formation of tumor nodules were determined by *in vivo* extravasation assay (A) and tumor metastasis assay (B), respectively. DiI staining of HT-29 cells (A) or SW480 cells (B) were transfected with 20 nM siNOX4, siANGPTL4, siMMP-1, and siMMP-9 and then injected intravenously into the tail vein of 6-week-old SCID-NOD mice which were preinjected intravenously with OA at a final concentration of 200 μM. To evaluate extravasation ability, mice were sacrificed up to 48 h after injection as described in 'Materials and Methods'. Tumor cell penetration was imaged using a microscope (A) (i). Original magnification, × 100; DiI labeled tumor cells (red); CD31

labeled blood vessels (green); DAPI labeled nucleus (blue). The number of tumor cell extravasation was calculated by analyzing at least four sections and six fields (A) (ii); Five mice were analyzed for each group. Real-time quantitative PCR analysis was performed to determine *NOX4* and *ANGPTL4* mRNA levels in cells treated with 200 μM OA for 48 h (A) (iii). Values are the mean ± SEM. *P*-values were determined using a two-tailed Student's *t*-test. ***$P < 0.001$. In addition, to evaluate lung metastasis, mice were sacrificed up to 6 weeks after injection as described in 'Materials and Methods'. H&E staining of lung tissue was imaged with a microscope (B) (i). The number of micronodules was counted under a microscope (B) (ii). Values are the mean ± SEM. *P*-values were determined using a two-tailed Student's *t*-test. **$P < 0.01$. ANGPTL4, NOX4, MMP-1, and MMP-9 expression were examined by IHC (B) (iii). n=5 for each group.

Figure S14. The expression of IL-6 and IL-8 is associated with ANGPTL4 and NOX4 levels. (A) Real-time quantitative PCR analysis of *IL-6* and *IL-8* mRNA levels was performed in SW480 cells treated with 200 μM OA for 24 h. The data are presented as the mean ± SEM. *P*-values were determined using a two-tailed Student's *t*-test. ***$P < 0.001$ (n=3). (B) Concurrent expression of ANGPTL4 and NOX4 comparing with IL-6 and IL-8 in tumor tissues of CRC patients (n=597) in TCGA database was quantitated (Pearson's correlation coefficient is shown in the figures). FPKM: Fragments Per Kilobase of transcript per Million.

References:

1. Chang KY, Shen MR, Lee MY, Wang WL, Su WC, Chang WC, et al. Epidermal growth factor-activated aryl hydrocarbon receptor nuclear translocator/HIF-1 signal pathway up-regulates cyclooxygenase-2 gene expression associated with squamous cell carcinoma. J Biol Chem. 2009; 284: 9908-16.
2. Chang WC, Wu SL, Huang WC, Hsu JY, Chan SH, Wang JM, et al. PTX3 gene activation in EGF-induced head and neck cancer cell metastasis. Oncotarget. 2015; 6: 7741-57.
3. Bai G, Hock TD, Logsdon N, Zhou Y, Thannickal VJ. A far-upstream AP-1/Smad binding box regulates human NOX4 promoter activation by transforming growth factor-beta. Gene. 2014; 540: 62-7.
4. Shen CJ, Chan SH, Lee CT, Huang WC, Tsai JP, Chen BK. Oleic acid-induced ANGPTL4 enhances head and neck squamous cell carcinoma anoikis resistance and metastasis via up-regulation of fibronectin. Cancer Lett. 2017; 386: 110-22.
5. Ko SC, Huang CR, Shieh JM, Yang JH, Chang WC, Chen BK. Epidermal growth factor protects squamous cell carcinoma against cisplatin-induced cytotoxicity through increased interleukin-1beta expression. PLoS One. 2013; e55795.
6. Lazzerini Denchi E, Celli G, de Lange T. Hepatocytes with extensive telomere deprotection and fusion remain viable and regenerate liver mass through endoreduplication. Genes Dev. 2006; 2648-53.

Figure S1

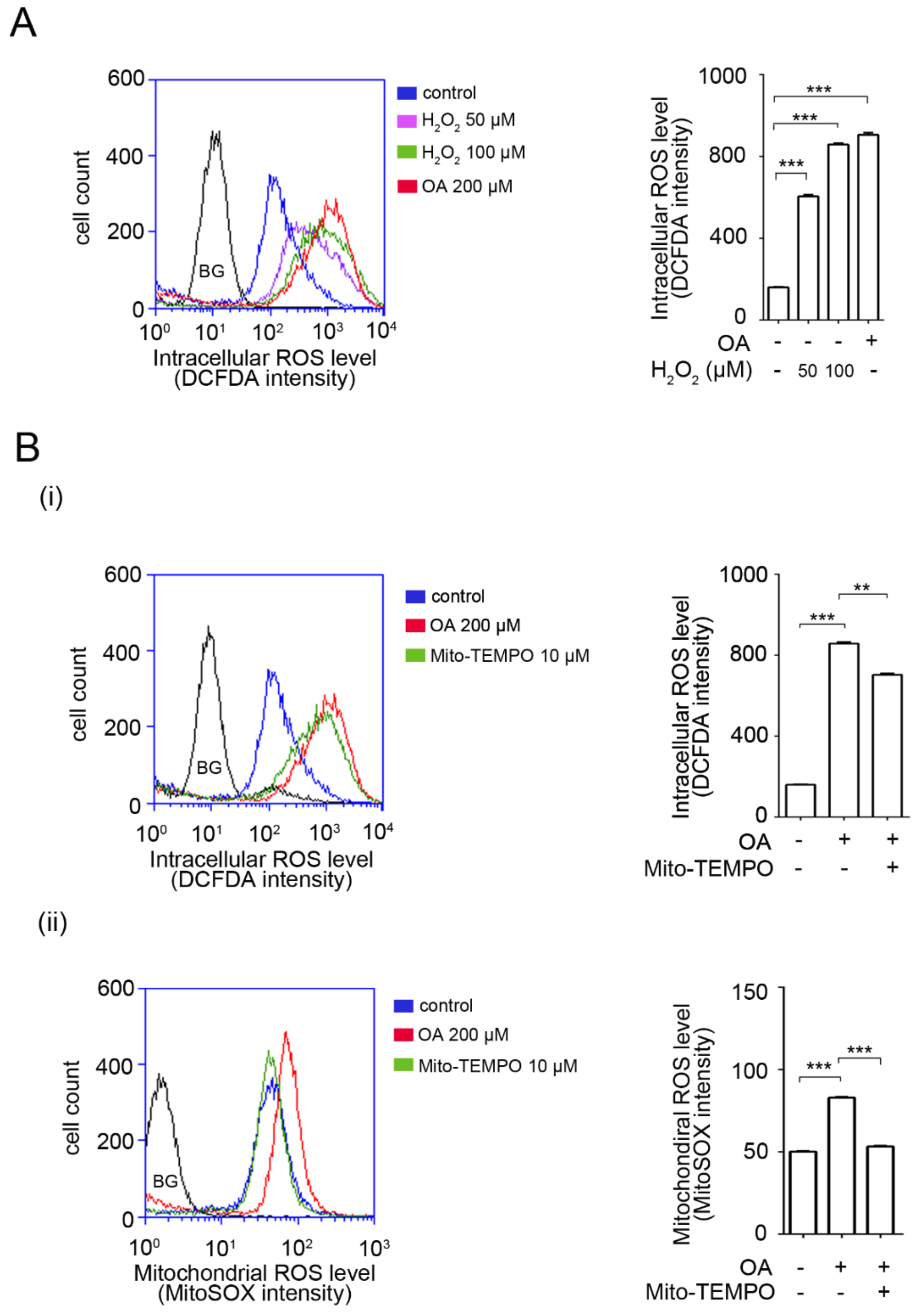
A
600
400
200
0
cell count
BG
10⁰ 10¹ 10² 10³ 10⁴
Intracellular ROS level (DCFDA intensity)
control
H2O2 50 μM
H2O2 100 μM
OA 200 μM
1000
800
400
0
Intracellular ROS level (DCFDA intensity)
***
***
***
OA - - - +
H2O2 (μM) - 50 100 -
B
(i)
600
400
200
0
cell count
BG
10⁰ 10¹ 10² 10³ 10⁴
Intracellular ROS level (DCFDA intensity)
control
OA 200 μM
Mito-TEMPO 10 μM
1000
800
400
0
Intracellular ROS level (DCFDA intensity)
***
**
OA - + +
Mito-TEMPO - - +
(ii)
600
400
200
0
cell count
BG
10⁰ 10¹ 10² 10³
Mitochondrial ROS level (MitoSOX intensity)
control
OA 200 μM
Mito-TEMPO 10 μM
150
100
50
0
Mitochondiral ROS level (MitoSOX intensity)
***
***
OA - + +
Mito-TEMPO - - +

Figure S2

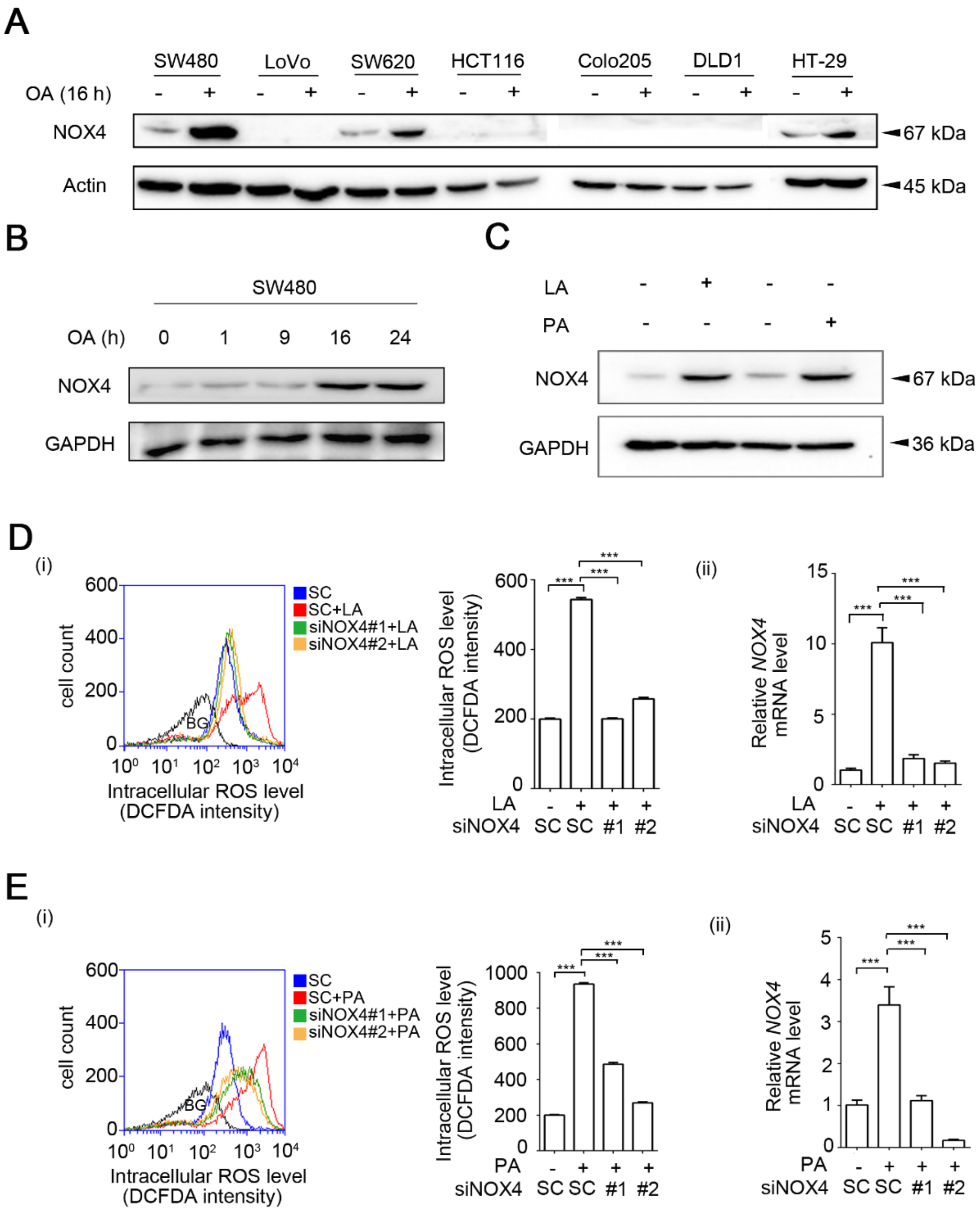
A
SW480
LoVo
SW620
HCT116
Colo205
DLD1
HT-29
OA (16 h)
NOX4
Actin
67 kDa
45 kDa
B
SW480
OA (h) 0 1 9 16 24
NOX4
GAPDH
C
LA - + - -
PA - - - +
NOX4
GAPDH
67 kDa
36 kDa
D
(i)
cell count
SC
SC+LA
siNOX4#1+LA
siNOX4#2+LA
BG
Intracellular ROS level (DCFDA intensity)
LA - + + +
siNOX4 SC SC #1 #2
(ii)
Relative NOX4 mRNA level
E
(i)
SC
SC+PA
siNOX4#1+PA
siNOX4#2+PA
cell count
Intracellular ROS level (DCFDA intensity)
PA - + + +
siNOX4 SC SC #1 #2
(ii)
Relative NOX4 mRNA level

Figure S3

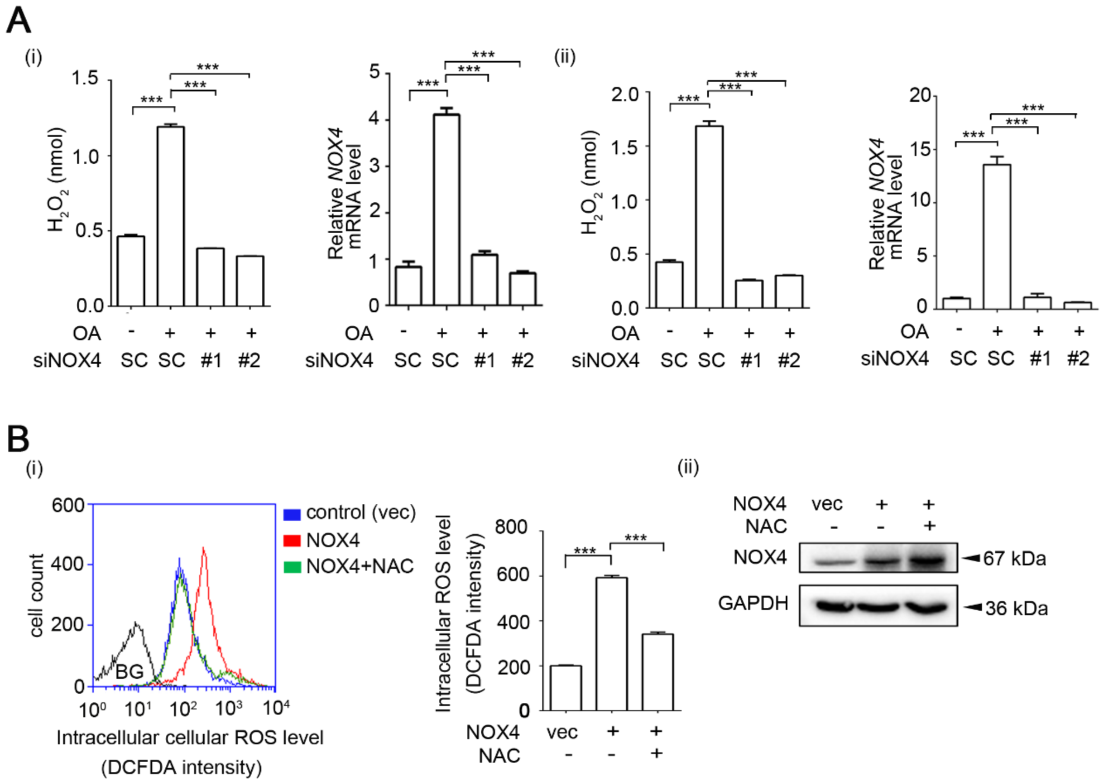
A
(i)
H2O2 (nmol)
Relative NOX4 mRNA level
OA - + + +
siNOX4 SC SC #1 #2
***
(ii)
B
(i)
cell count
control (vec)
NOX4
NOX4+NAC
BG
Intracellular cellular ROS level
(DCFDA intensity)
Intracellular ROS level
(DCFDA intensity)
NOX4 vec + +
NAC - - +
(ii)
NOX4
GAPDH
67 kDa
36 kDa

Figure S4

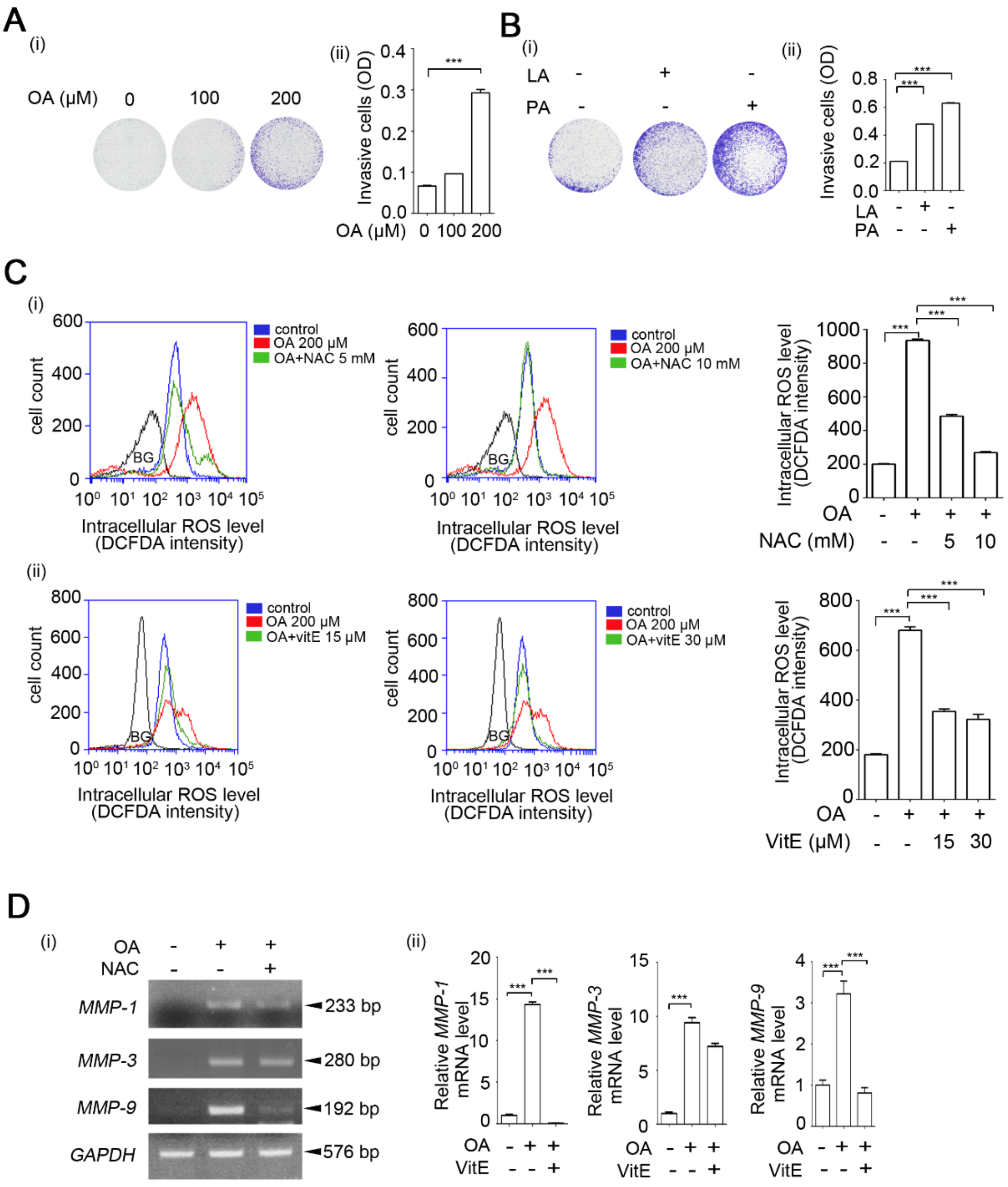
A
(i)
OA (μM) 0 100 200
(ii)
Invasive cells (OD)
OA (μM) 0 100 200
B
(i)
LA - + -
PA - - +
(ii)
Invasive cells (OD)
LA - + -
PA - - +
C
(i)
cell count
control
OA 200 μM
OA+NAC 5 mM
OA+NAC 10 mM
BG
Intracellular ROS level (DCFDA intensity)
OA - + + +
NAC (mM) - - 5 10
(ii)
control
OA 200 μM
OA+vitE 15 μM
OA+vitE 30 μM
OA - + + +
VitE (μM) - - 15 30
D
(i)
OA - + +
NAC - - +
MMP-1 233 bp
MMP-3 280 bp
MMP-9 192 bp
GAPDH 576 bp
(ii)
Relative MMP-1 mRNA level
Relative MMP-3 mRNA level
Relative MMP-9 mRNA level
OA - + +
VitE - - +

Figure S5

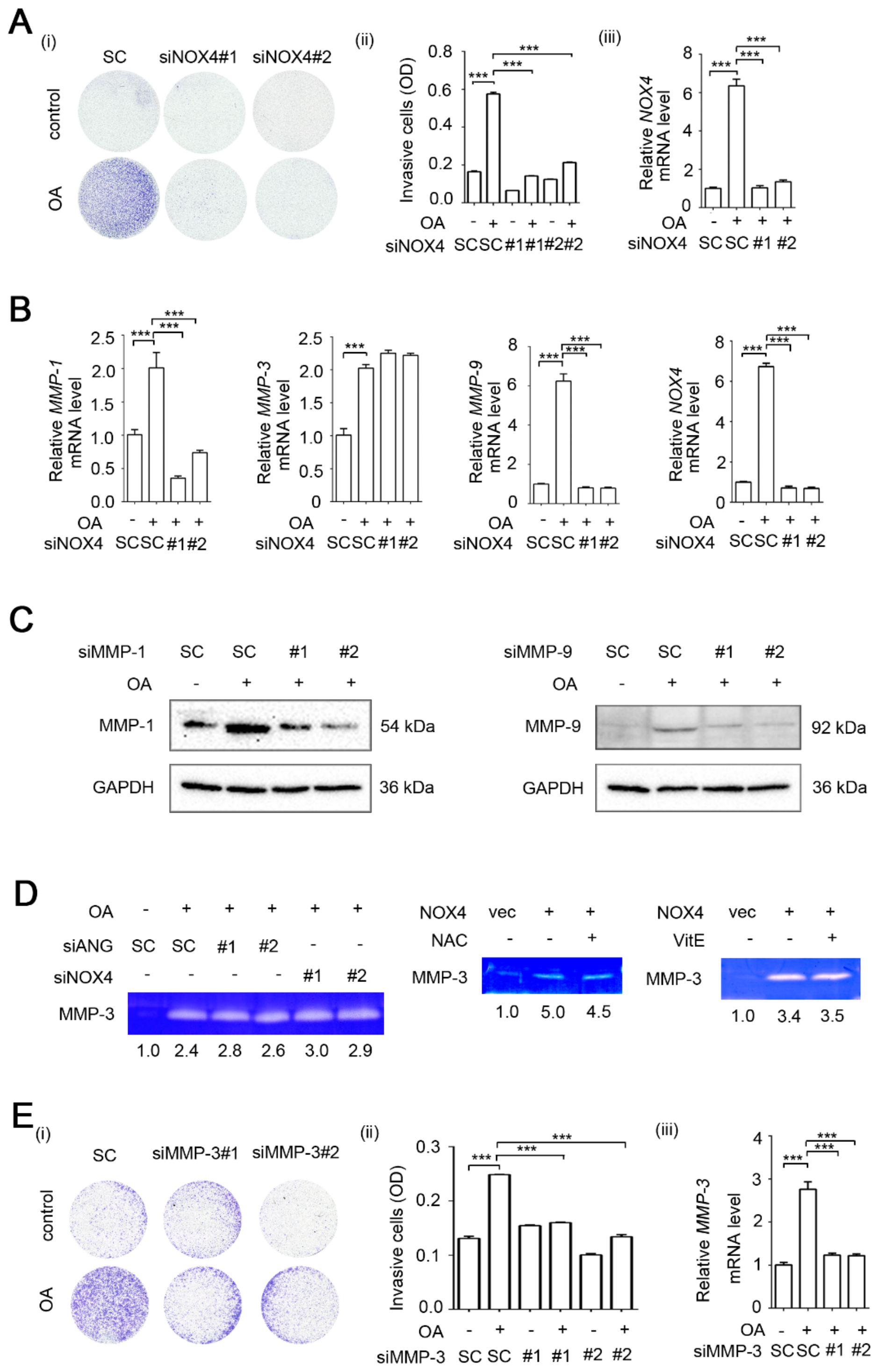

A
(i)
SC
siNOX4#1
siNOX4#2
control
OA
(ii)
Invasive cells (OD)
OA
siNOX4
SC SC #1 #1 #2 #2
(iii)
Relative NOX4 mRNA level
siNOX4
SC SC #1 #2
B
Relative MMP-1 mRNA level
Relative MMP-3 mRNA level
Relative MMP-9 mRNA level
Relative NOX4 mRNA level
OA
siNOX4
SC SC #1 #2
C
siMMP-1
SC SC #1 #2
OA
MMP-1
54 kDa
GAPDH
36 kDa
siMMP-9
SC SC #1 #2
MMP-9
92 kDa
GAPDH
36 kDa
D
OA
siANG
SC SC #1 #2
siNOX4
#1 #2
MMP-3
1.0 2.4 2.8 2.6 3.0 2.9
NOX4
vec
NAC
MMP-3
1.0 5.0 4.5
NOX4
vec
VitE
MMP-3
1.0 3.4 3.5
E
(i)
SC
siMMP-3#1
siMMP-3#2
control
OA
(ii)
Invasive cells (OD)
OA
siMMP-3
SC SC #1 #1 #2 #2
(iii)
Relative MMP-3 mRNA level
OA
siMMP-3
SC SC #1 #2

Figure S6

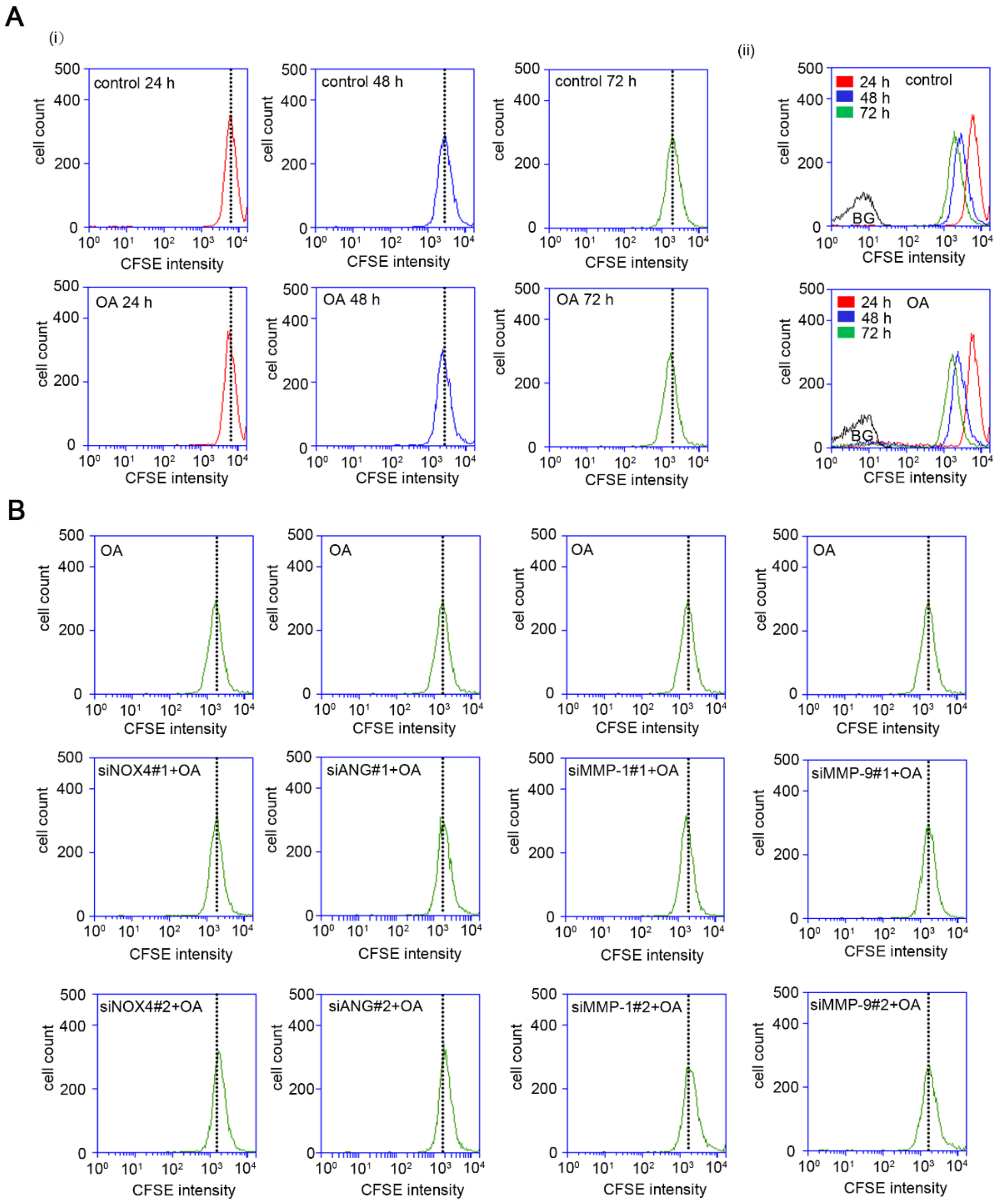
A
(i)
control 24 h
control 48 h
control 72 h
OA 24 h
OA 48 h
OA 72 h
(ii)
24 h
48 h
72 h
control
OA
BG
cell count
CFSE intensity
B
OA
siNOX4#1+OA
siANG#1+OA
siMMP-1#1+OA
siMMP-9#1+OA
siNOX4#2+OA
siANG#2+OA
siMMP-1#2+OA
siMMP-9#2+OA

Figure S7

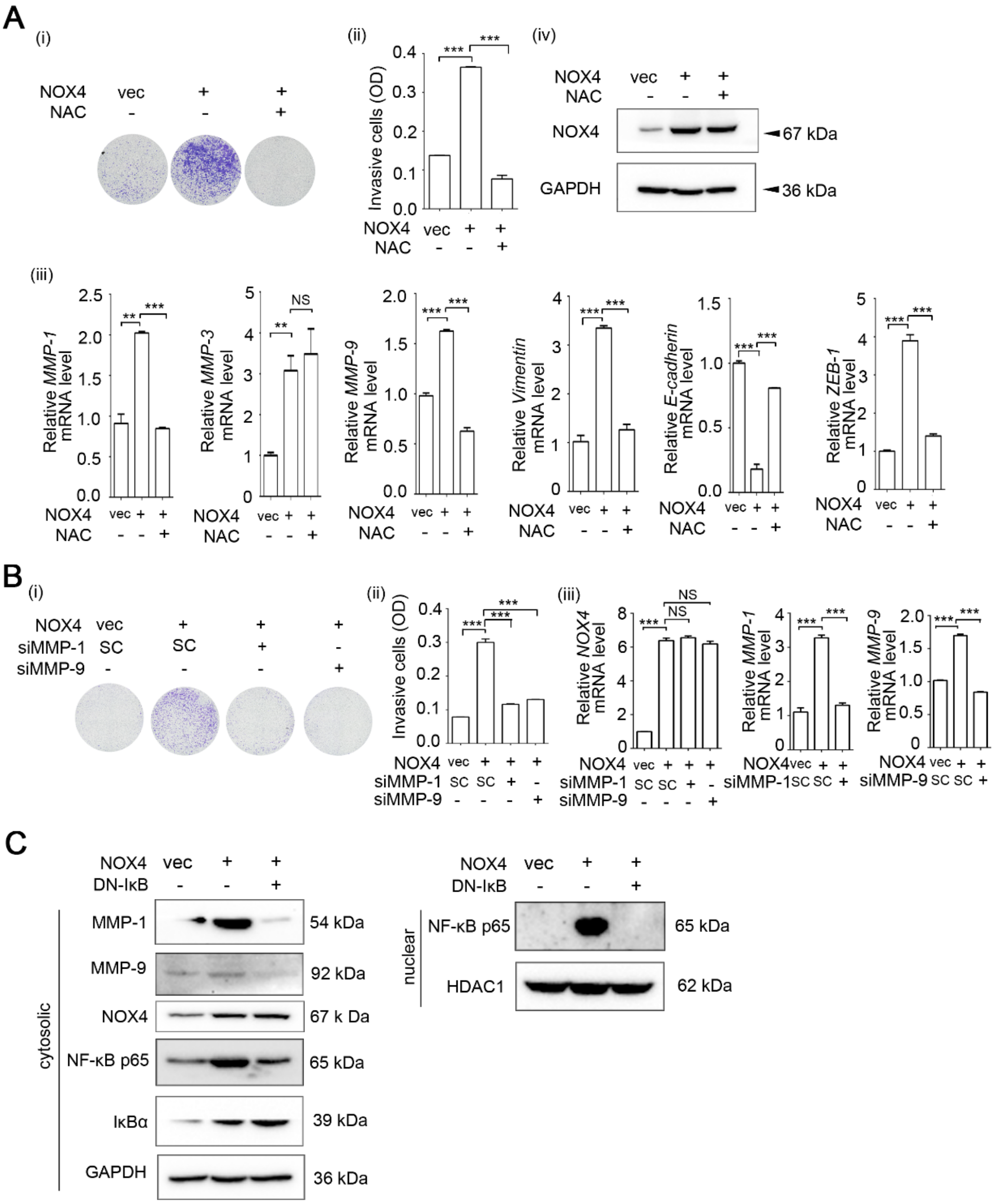

A
(i)
NOX4 vec + +
NAC - - +
(ii)
Invasive cells (OD)
NOX4 vec + +
NAC - - +
(iv)
NOX4 vec + +
NAC - - +
NOX4
67 kDa
GAPDH
36 kDa
(iii)
Relative MMP-1 mRNA level
Relative MMP-3 mRNA level
Relative MMP-9 mRNA level
Relative Vimentin mRNA level
Relative E-cadherin mRNA level
Relative ZEB-1 mRNA level
NOX4 vec + +
NAC - - +
NS
B
(i)
NOX4 vec + + +
siMMP-1 SC SC + -
siMMP-9 - - - +
(ii)
Invasive cells (OD)
(iii)
Relative NOX4 mRNA level
Relative MMP-1 mRNA level
Relative MMP-9 mRNA level
NS
C
NOX4 vec + +
DN-IκB - - +
cytosolic
MMP-1 54 kDa
MMP-9 92 kDa
NOX4 67 k Da
NF-κB p65 65 kDa
IκBα 39 kDa
GAPDH 36 kDa
nuclear
NF-κB p65 65 kDa
HDAC1 62 kDa

Figure S8

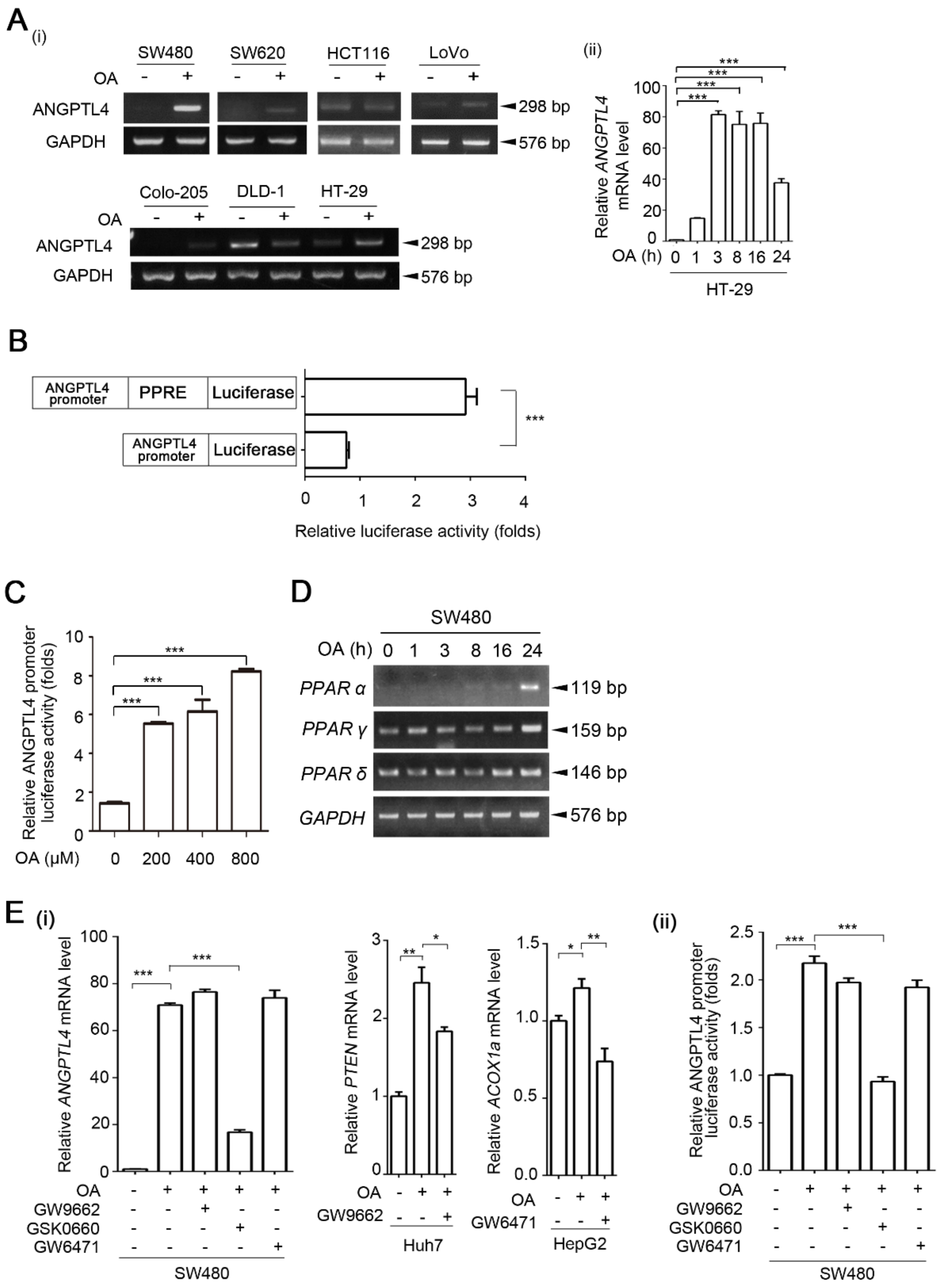
A (i)
SW480
SW620
HCT116
LoVo
OA
ANGPTL4
GAPDH
298 bp
576 bp
Colo-205
DLD-1
HT-29
(ii)
Relative ANGPTL4 mRNA level
OA (h) 0 1 3 8 16 24
HT-29
B
ANGPTL4 promoter
PPRE
Luciferase
Relative luciferase activity (folds)
C
Relative ANGPTL4 promoter luciferase activity (folds)
OA (μM) 0 200 400 800
D
SW480
OA (h) 0 1 3 8 16 24
PPAR α
PPAR γ
PPAR δ
GAPDH
119 bp
159 bp
146 bp
576 bp
E (i)
Relative ANGPTL4 mRNA level
OA
GW9662
GSK0660
GW6471
SW480
Relative PTEN mRNA level
Huh7
Relative ACOX1a mRNA level
HepG2
(ii)
Relative ANGPTL4 promoter luciferase activity (folds)

Figure S9

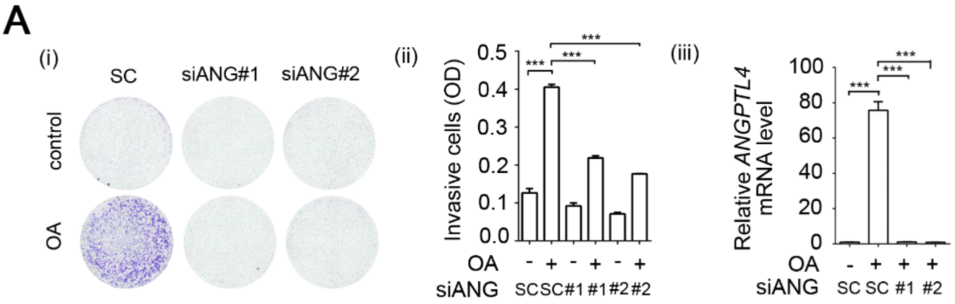

A
(i)
SC
siANG#1
siANG#2
control
OA
(ii)
Invasive cells (OD)
0.5
0.4
0.3
0.2
0.1
0.0
***
***
***
OA - + - + - +
siANG SC SC #1 #1 #2 #2
(iii)
Relative ANGPTL4 mRNA level
100
80
60
40
20
0
***
***
***
OA - + + +
siANG SC SC #1 #2

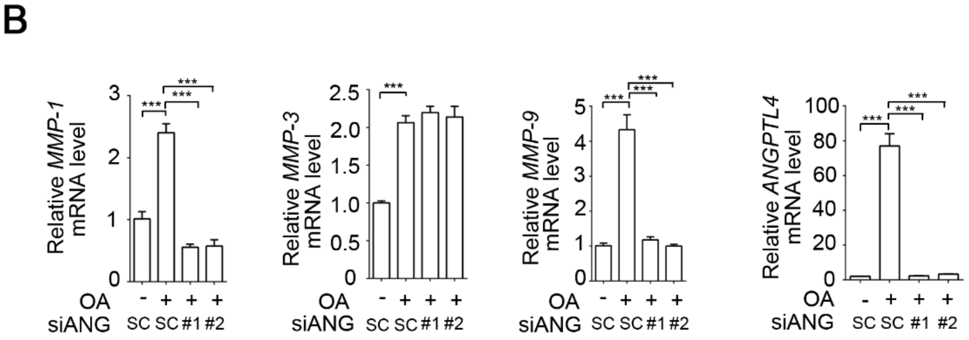

B
Relative MMP-1 mRNA level
3
2
1
0
***
***
***
OA - + + +
siANG SC SC #1 #2
Relative MMP-3 mRNA level
2.5
2.0
1.5
1.0
0.5
0
***
OA - + + +
siANG SC SC #1 #2
Relative MMP-9 mRNA level
5
4
3
2
1
0
***
***
***
OA - + + +
siANG SC SC #1 #2
Relative ANGPTL4 mRNA level
100
80
60
40
20
0
***
***
***
OA - + + +
siANG SC SC #1 #2

Figure S10

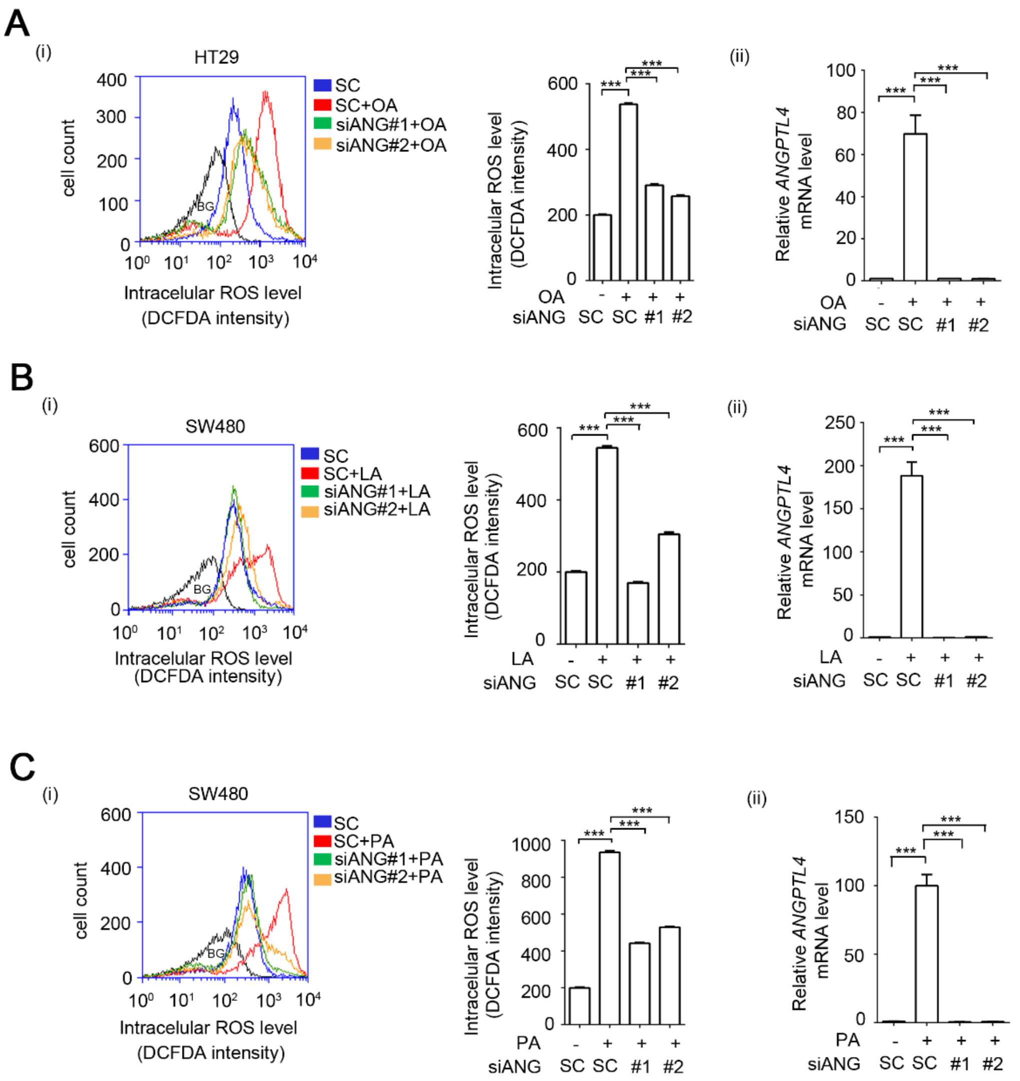

A
(i)
HT29
SC
SC+OA
siANG#1+OA
siANG#2+OA
cell count
Intracelular ROS level
(DCFDA intensity)
BG
Intracelular ROS level
(DCFDA intensity)
OA
siANG
SC SC #1 #2
(ii)
Relative ANGPTL4
mRNA level
***
B
(i)
SW480
SC
SC+LA
siANG#1+LA
siANG#2+LA
cell count
Intracelular ROS level
(DCFDA intensity)
LA
siANG
SC SC #1 #2
(ii)
Relative ANGPTL4
mRNA level
C
(i)
SW480
SC
SC+PA
siANG#1+PA
siANG#2+PA
cell count
Intracelular ROS level
(DCFDA intensity)
PA
siANG
SC SC #1 #2
(ii)
Relative ANGPTL4
mRNA level

Figure S11

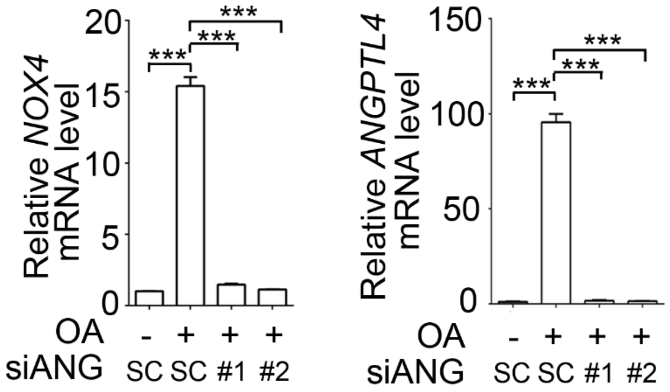

20
15
10
5
0
Relative NOX4 mRNA level
***
***
***
OA
siANG
-
+
+
+
SC
SC
#1
#2
150
100
50
0
Relative ANGPTL4 mRNA level
***
***
***
OA
siANG
-
+
+
+
SC
SC
#1
#2

Figure S12

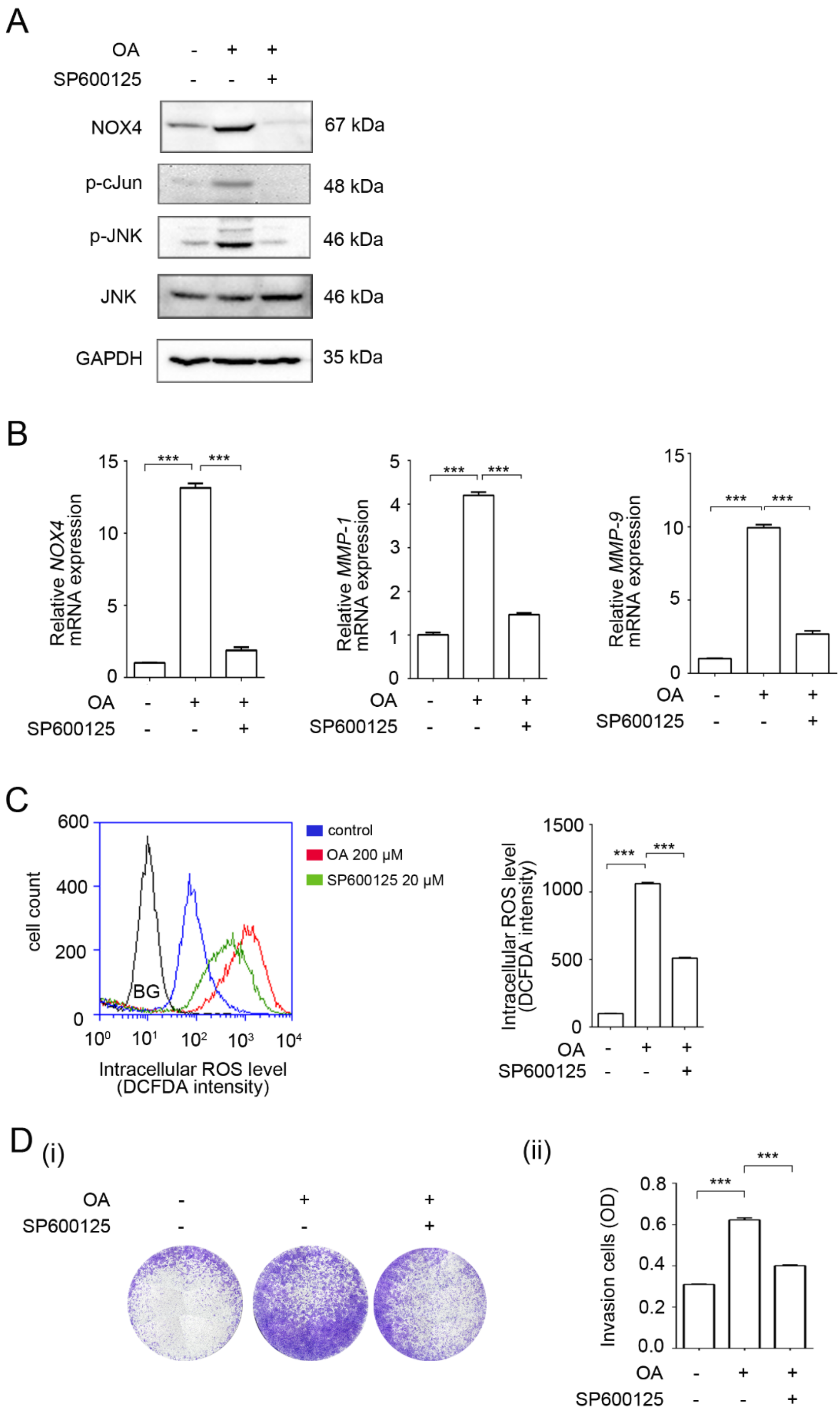
A
OA - + +
SP600125 - - +
NOX4 67 kDa
p-cJun 48 kDa
p-JNK 46 kDa
JNK 46 kDa
GAPDH 35 kDa
B
Relative NOX4 mRNA expression
Relative MMP-1 mRNA expression
Relative MMP-9 mRNA expression
***
OA - + +
SP600125 - - +
C
cell count
control
OA 200 μM
SP600125 20 μM
BG
Intracellular ROS level (DCFDA intensity)
Intracellular ROS level (DCFDA intensity)
OA - + +
SP600125 - - +
D (i)
OA - + +
SP600125 - - +
(ii)
Invasion cells (OD)
OA - + +
SP600125 - - +

Figure S13

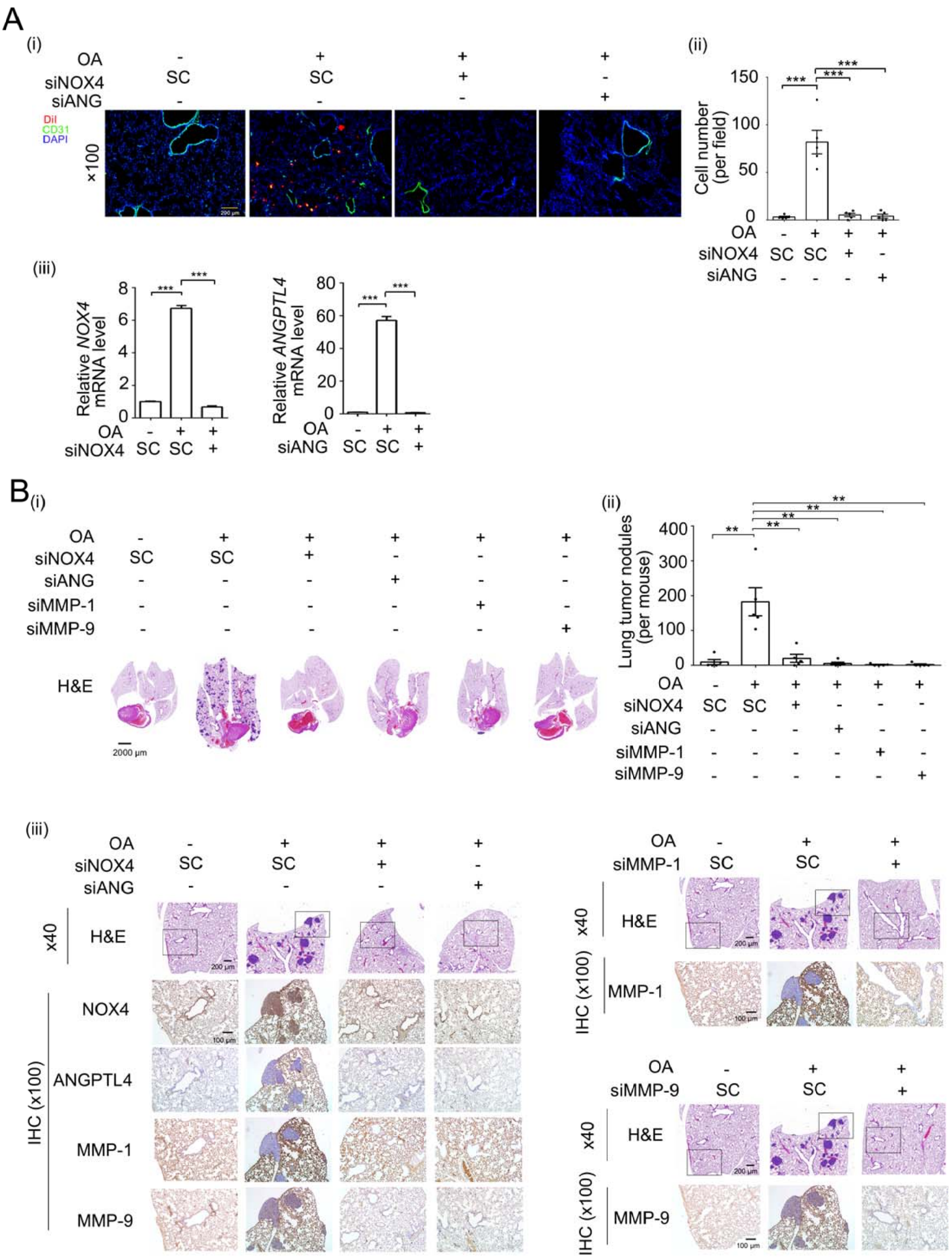
A
(i)
OA - + + +
siNOX4 SC SC + -
siANG - - - +
DiI
CD31
DAPI
×100
(ii)
Cell number (per field)
150
100
50
0
***
***
***
OA - + + +
siNOX4 SC SC + -
siANG - - - +
(iii)
Relative NOX4 mRNA level
8
6
4
2
0
***
***
OA - + +
siNOX4 SC SC +
Relative ANGPTL4 mRNA level
80
60
40
20
0
***
***
OA - + +
siANG SC SC +
B
(i)
OA - + + + + +
siNOX4 SC SC + - - -
siANG - - - + - -
siMMP-1 - - - - + -
siMMP-9 - - - - - +
H&E
2000 μm
(ii)
Lung tumor nodules (per mouse)
400
300
200
100
0
**
**
**
**
**
OA - + + + + +
siNOX4 SC SC + - - -
siANG - - - + - -
siMMP-1 - - - - + -
siMMP-9 - - - - - +
(iii)
OA - + + +
siNOX4 SC SC + -
siANG - - - +
x40
H&E
200 μm
IHC (x100)
NOX4
100 μm
ANGPTL4
MMP-1
MMP-9
OA - + +
siMMP-1 SC SC +
x40
H&E
200 μm
IHC (x100)
MMP-1
100 μm
OA - + +
siMMP-9 SC SC +
x40
H&E
200 μm
IHC (x100)
MMP-9
100 μm

Figure S14

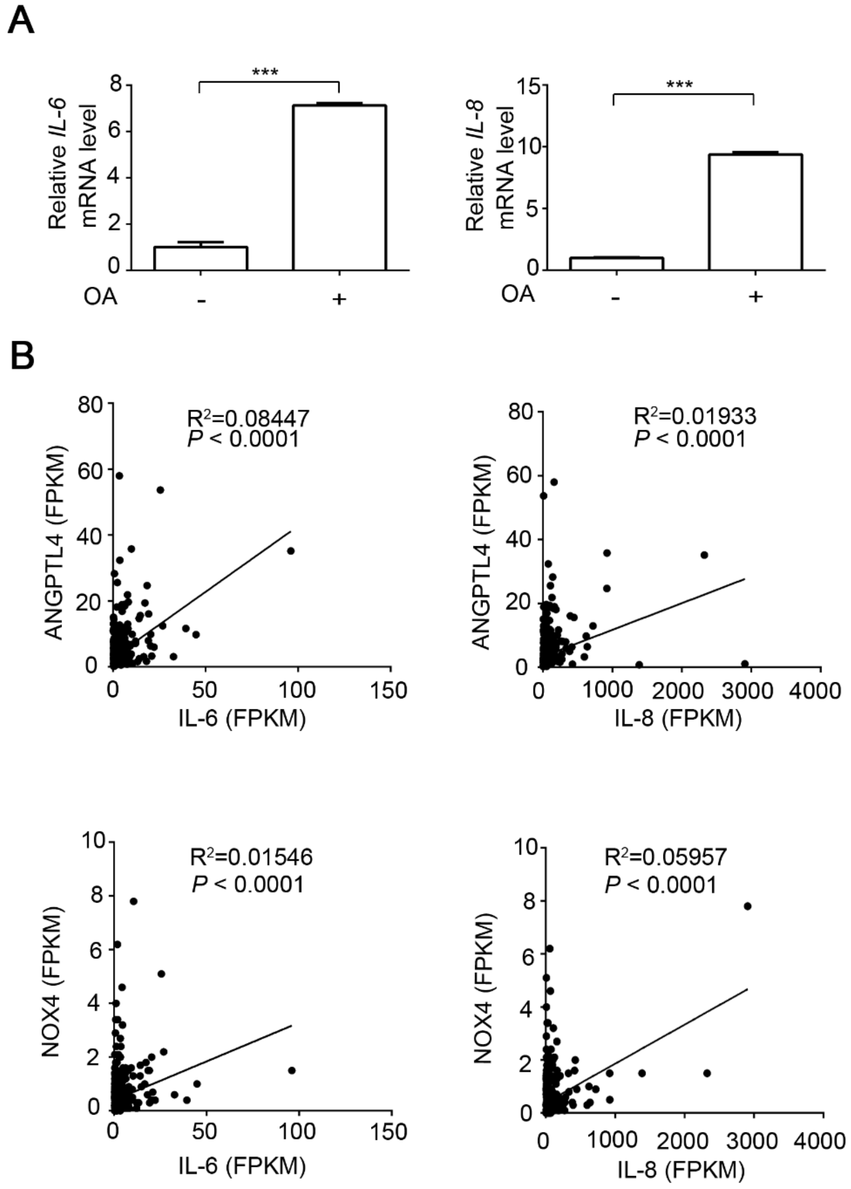

A
8
6
4
2
0
Relative IL-6 mRNA level
***
OA
-
+
15
10
5
0
Relative IL-8 mRNA level
***
OA
-
+
B
80
60
40
20
0
ANGPTL4 (FPKM)
R2=0.08447
P < 0.0001
0
50
100
150
IL-6 (FPKM)
80
60
40
20
0
ANGPTL4 (FPKM)
R2=0.01933
P < 0.0001
0
1000
2000
3000
4000
IL-8 (FPKM)
10
8
6
4
2
0
NOX4 (FPKM)
R2=0.01546
P < 0.0001
0
50
100
150
IL-6 (FPKM)
10
8
6
4
2
0
NOX4 (FPKM)
R2=0.05957
P < 0.0001
0
1000
2000
3000
4000
IL-8 (FPKM)
